# Identification of *Plasmodium* sporozoites using angle-resolved scattered light pulse shapes

**DOI:** 10.1371/journal.pone.0354060

**Published:** 2026-11-05

**Authors:** Vinod Devaraj, Sophia Kahler, Daniel Kage, Elena A. Levashina, Toralf Kaiser

**Affiliations:** 1 Flow Cytometry Core Facility, German Rheumatology Research Center (DRFZ), Berlin, Germany; 2 Vector Biology Research Unit, Max Planck Institute for Infection Biology (MPIIB), Berlin, Germany; Freie Universität Berlin, GERMANY

## Abstract

Flow cytometry is widely used to identify and sort rare cells, but conventional approaches rely on fluorescent labels. Label-free discrimination of rare targets in complex, heterogeneous samples remains challenging. Here, we introduce a label-free strategy that combines multi-angle pulse shape flow cytometry (MAPS-FC), which exploits the full temporal structure of the signal pulses during cell or particles transit through the laser spot, with a deep learning–based analysis. As a biologically relevant test case, we focus on *Plasmodium falciparum* sporozoites, the infectious stage of the malaria parasite, which need to be isolated from mosquito salivary glands and are embedded in a heterogeneous mosquito-derived background. Using a GFP-expressing reporter line for validation, we trained a long short-term memory (LSTM) neural network to classify sporozoites solely from their scattering pulse shapes captured through MAPS-FC. Using training data from multiple days and setting a threshold on the classification confidence, the network can distinguish sporozoites from mosquito debris without fluorescent labels. This work establishes MAPS-FC with deep learning as a platform for label-free identification of rare parasites in complex biological samples.

## 1. Introduction

Flow cytometry is a well-established technique for cell analysis and sorting, but it predominantly relies on fluorescent labels to identify cells or particles of interest [1]. Although label-free approaches based on light scattering have gained attention [2–4], robust discrimination of rare biological targets within complex and heterogeneous samples remains a major challenge.

We previously developed a flow-cytometric method that employs time- and angle-resolved detection of the scattered light: multi-angle pulse shape flow cytometry (MAPS-FC) [5–7]. This approach incorporates a specialized flow cytometry setup that captures scattered light pulse shapes from multiple scattering angles, extending beyond the standard forward and side scatter measurements. The technique has proven feasible for cell cycle analysis [5–7] and was validated through comparison with mathematical simulations of light scattering [8]. However, its performance on rare targets within complex, heterogeneous backgrounds has not yet been demonstrated.

Here, we address this challenge by applying MAPS-FC to *Plasmodium falciparum* sporozoites, the infectious stage of the malaria parasite, as a biologically relevant test system. Sporozoite development within the mosquito host takes roughly 2–3 weeks. Although mosquito-free methods to develop *P. falciparum* sporozoites have been reported [9,10], this parasite stage cannot currently be cultured reliably at large scale *in vitro* [11]. Instead, they are typically dissected from the salivary glands of infected mosquitoes, yielding preparations that are intrinsically heterogeneous with a large excess of mosquito-derived cells and debris [12–14]. Flow cytometry is a promising platform for analyzing sporozoites due to its high throughput, and its ability to maintain parasite viability [13,15,16]. Nevertheless, conventional flow cytometric approaches typically rely on fluorescent antibody labeling, nuclear staining or genetically engineered reporter lines expressing fluorescent proteins to distinguish sporozoites from mosquito debris [17–20]. Label-free identification has been reported for certain *Plasmodium* species using specific commercially available flow cytometers, but these methods often suffer from limited purity or require highly optimized sample preparation [21,22]. Therefore, we examined whether the high-dimensional MAPS-FC data combined with deep neural networks enables label-free classification of *P. falciparum* sporozoites within complex mosquito-derived samples.

Using a *P. falciparum* reporter line [20] which expresses green fluorescent protein (GFP) for validation, we trained and validated a long short-term memory (LSTM)–based neural network capable of identifying sporozoites solely from their scattering pulse shapes. LSTM exploits the full temporal structure of the measured pulses captured by MAPS-FC. Thereby it enables detection of subtle, distributed differences in pulse dynamics that are not easily accessible with conventional feature-based classifiers. Different modalities in data analysis, such as the combination of training data from multiple experiments and thresholds on the classification confidence further improved classification precision. Together, this work establishes MAPS-FC with deep learning as a platform for label-free parasite identification in complex biological samples.

## 2. Methods

### 2.1. Sporozoite preparation

#### 2.1.1. Parasite cultures.

*Pf*OBC13^GFP^ NF54 *P. falciparum* parasites [20] were cultured in the lab as previously described by *Siciliano et al.*[23]. Parasites were grown in 0^+^ human red blood cells (Haema, Berlin), at 37 °C, under a 3% O_2_, 5% CO_2_, and 92% N_2_ atmosphere. Asexual cultures (0.5–5% of parasitemia) were kept at 3–4% hematocrit in complete RPMI 1640 medium containing L-glutamine and 25 mM HEPES (Thermo Fisher Scientific), supplemented with 10% human A+ serum (Haema, Berlin), 10 mM hypoxanthine (c-c-Pro), and 20 µg/mL of gentamicin (Sigma). *P. falciparum* asexual cultures were routinely screened for *Mycoplasma* contamination on a monthly basis. For gametocyte induction, asynchronous asexual parasites were seeded at 1% parasitemia and 5% hematocrit. Cultures were maintained for 15 days with daily replacement of complete medium without gentamicin and were then used for mosquito infection experiments. Stage V gametocytes were quantified 14–15 days post-seeding under the microscope using a Malassez counting chamber.

#### 2.1.2. Mosquito rearing and parasite infection.

*Anopheles coluzzii* (Ngousso S1 strain) mosquitoes were reared at 28°C under 70–80% humidity with a 12/12-h day/night cycle. For *Pf*OBC13^GFP^ infections, mosquitoes were fed for 15 min via a membrane feeding system containing stage V gametocytes adjusted to ~10⁷ gametocytes/mL in fresh red blood cells and human serum at a final hematocrit of 50%. Infected mosquitoes were maintained in a secured Biosafety Level 3 (S3) facility at 27°C in compliance with national regulations (Landesamt für Gesundheit und Soziales, project number 297/13). Unfed mosquitoes were discarded shortly after the feeding procedure. Eight days after gametocyte infection, the infection status of blood-fed mosquitoes was assessed by midgut oocyst counting. Across experiments, 90–100% of mosquitoes were infected.

#### 2.1.3. Mosquito salivary gland dissection and preparation for flow cytometry.

*A. coluzzii* mosquitoes were killed in ethanol 14–15 days post-infection in a Biosafety Level 3 (S3) laboratory. A total of 30–120 mosquito salivary glands were dissected under the stereoscope in RPMI 1640 with L-glutamine and 25 mM HEPES (Thermo Fisher Scientific) supplemented with 3% bovine serum albumin (BSA) (Biomol). Dissection involved mosquito head-cutting and extracting the salivary glands along with associated mosquito material from the upper thorax. The upper thoracic region was excised, collected into a grinding tube, homogenized manually using a glass grinder and subsequently filtered through a 40 µm nylon cell strainer (Falcon) into a glass tube. The total volume of the filtered extract was measured, and the volume per dissected mosquito material was calculated. For subsequent analysis, the volume corresponding to three mosquito equivalents was diluted in 200 µL of phosphate-buffered saline (PBS). The overall preparation time amounts to approximately 1–2 h depending on the number of mosquitoes. Salivary gland sporozoite numbers averaged 20,000–60,000 sporozoites per mosquito per experiment (overall average ~34,000 sporozoites per mosquito). The sample was placed on ice until flow cytometry analysis, which was performed within one hour of preparation.

### 2.2. Flow cytometry and scattered light detection

Samples containing sporozoites along with mosquito debris were analyzed with a custom-built flow cytometer with advanced scattered light detection capabilities [6,7]. This setup encompasses optical fiber arrays for the detection of forward and sideward scattered (FSC/SSC) light at multiple angles within the main directions. The optical setup is outlined in Fig 1.

**Fig 1:**
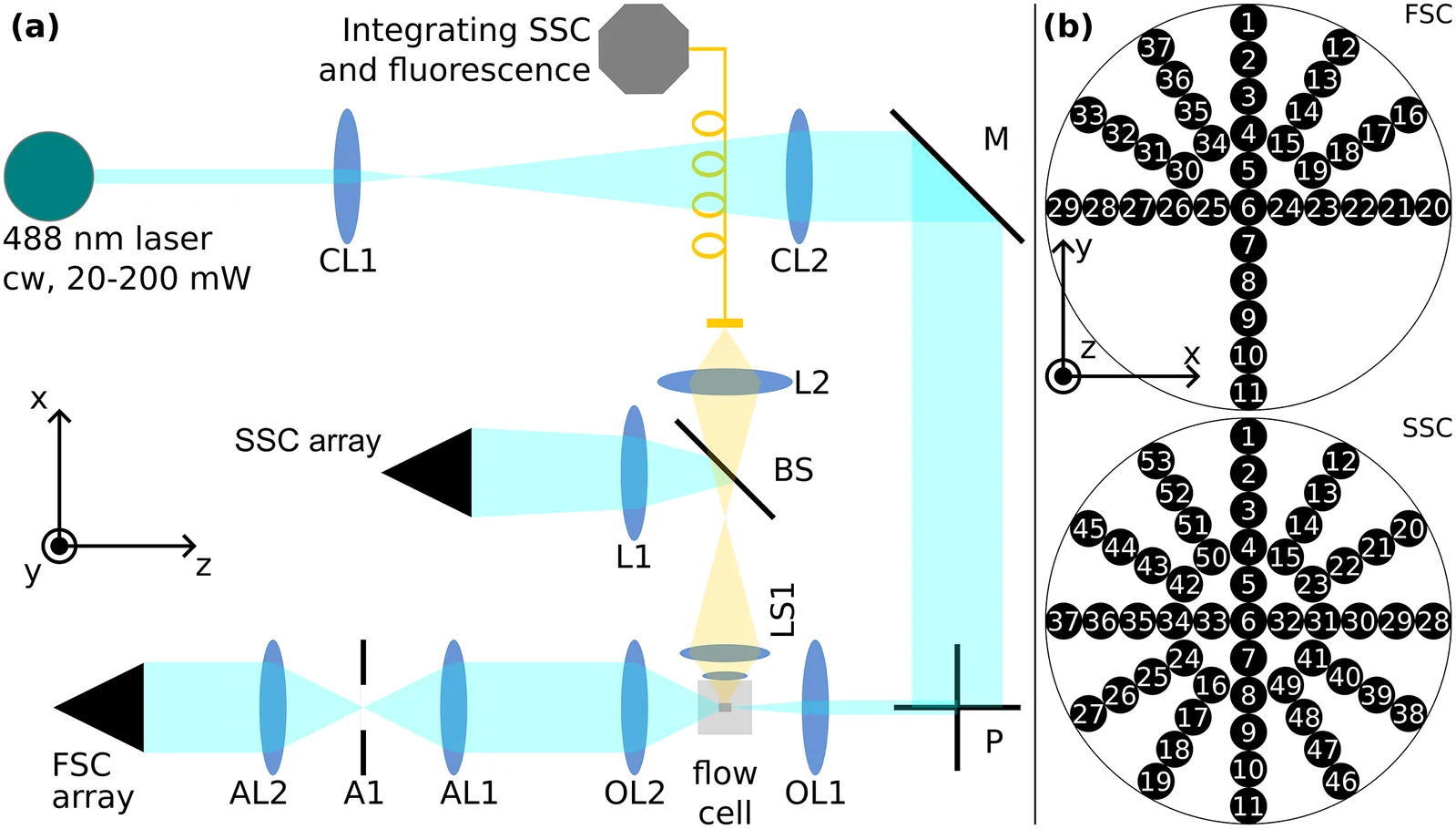
The optical setup. (a) Schematic drawing of a top view on the optical setup of the custom-built flow cytometer. (b) Layout of the arrays of optical fibers for FSC and SSC light collection. The coordinate system indicates the orientation with respect to the setup. A detailed listing of the components is given in Supporting Information Section 1.

A 488-nm continuous wave laser (Coherent, Santa Clara, USA) set to 20 mW optical output power is used as the light source. The beam is shaped by a cylindrical telescope (CL1 & 2), and then directed (M, P) through an objective (OL1) which focuses the beam into the flow cell cuvette. The estimated 1/e^2^ half width in the focal point amounts to 3 µm along the flow direction of the particles. The channel cross section in the flow cuvette is rectangular with dimensions 480x180 µm. Forward scattered light is collected with an objective (OL2) and passed through a slit aperture (AL1, A1, AL2). Side scattered light and fluorescence are collected with a lens system (LS). A beam splitter (BS) directs a large fraction of light with 488 nm wavelength to the SSC array (L1). The residual light is focused (L2) onto a fiber connected to a PMT holder with dichroic mirrors and spectral filters for integrating SSC and fluorescence detection. For scattered light detection, bandpasses with 488 nm central wavelength and 10 nm spectral width were used. The GFP fluorescence was detected with 530/30 nm bandpass. Depending on the respectively selected fibers in the detector arrays, neutral density filters were used to operate the PMTs at appropriate voltages. A comprehensive listing of all optical components can be found in the Supporting Information Section 1. During cell or particle transit through the laser beam, full signal pulses are acquired for further analysis. A signal processing system (APE, Berlin, Germany) was employed to digitize the signals at 25 MHz sampling rate and a digital resolution of 16 bit in 8 channels. For the measurements performed, the 8 channels were divided into 7 light scatter channels and 1 fluorescence channel. Data acquisition for an event is started by a threshold trigger on the first channel (FSC23) with a pre-trigger region on 0.24 µs.

### 2.3. Data analysis

The pulse shape data is a sequential input, where each pulse measures the temporal scattering profile of a particle in a certain angular range as it passes through the laser focus. To correctly classify parasite and non-parasite events, it is important to not only analyze the pulse shape at each time step but also to capture the temporal dependencies across the entire pulse duration. This allows the model to learn the sequence as well as the relationships between the adjacent time points in the pulse shape.

Long Short-Term Memory (LSTM) networks are well-suited to model this type of sequential data [24–26]. Using a gating mechanism with forget gates, input gates, and output gates, LSTM networks can remember or forget selectively, allowing learning of both short-range and long-range dependencies in the pulse shapes. This resolves the disappearing and exploding gradients issues which occur in recurrent neural networks (RNNs) [27]. LSTM networks have been employed to capture temporal dependencies by propagating information from the past time points in a pulse form. But in order to gain a more general perspective of the temporal information, it may be helpful to incorporate information from future as well as past time points. In such cases, a Bidirectional Long Short-Term Memory (BiLSTM) network is utilized to observe two-way relations across the pulse shape [28]. BiLSTM accomplishes this by combining a forward LSTM, reading the pulse from the start to finish, and a backward LSTM, reading it in the reverse order.

The proposed model used for classification of sporozoites and mosquito host cells are based on pulse shapes and their first derivative values observed across different light scattering angles. It is represented schematically in Fig 2. It has two separate branches; one of them processes raw pulse of shape (4, 80), where 4 and 80 indicate the number of detectors and the number of data points in each pulse. This is then fed into the BiLSTM layer of 100 units (units correspond to memory cells) set to return sequences. Then the BiLSTM layer output is passed to a unidirectional LSTM layer with 50 units. The unidirectional LSTM projects the sequence into one vector. Both the layers include a dropout and recurrent dropout, both 0.2, in order to control overfitting and model generalization. Meanwhile, the second branch of LSTM handles only the first derivatives of the pulse shapes. This branch is identical to the first branch which enables the model to capture both temporal aspects of the pulse shapes and their rate of change.

**Fig 2:**
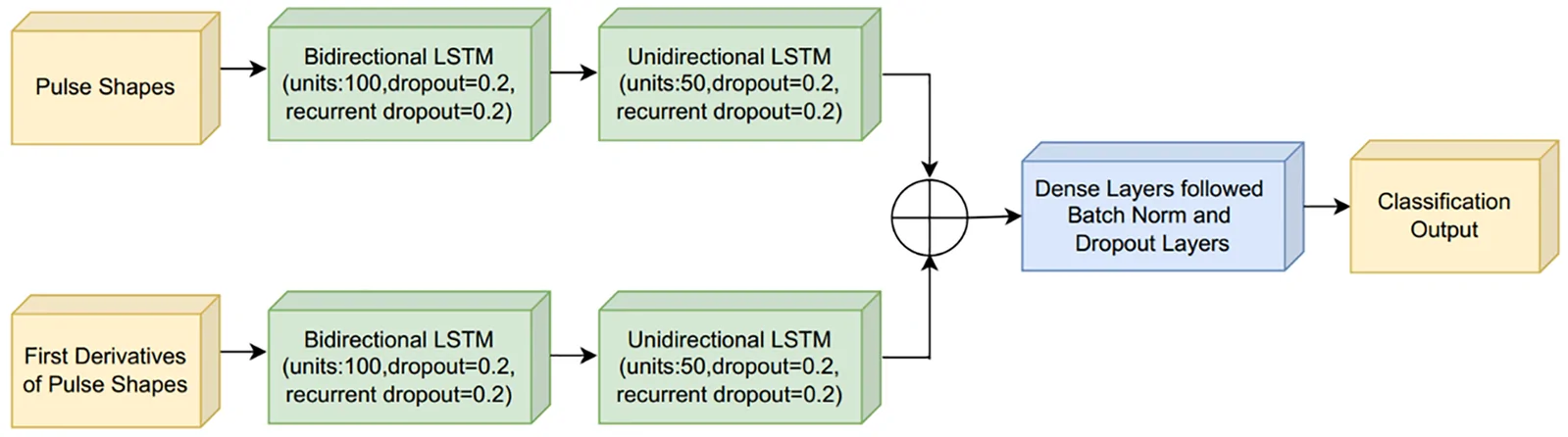
Bi-directional LSTM-based AI model.

The last representation is formed by concatenation of outputs of the two LSTM branches into a single vector embedding information from both pulse shape as well as their derivatives. The combined vector is then projected by a dense layer with 50 units and ReLU activation function to facilitate learning complex non-linear relations in data [29,30]. Additionally, after the primary convolutional body, batch normalization is used to stabilize the training. Furthermore, a 0.5 rate dropout layer is added in order to minimize overfitting. A final classification layer is dense with softmax activation and produces probabilities for the two classes. The model is trained with an Adam optimizer and sparse categorical cross-entropy loss [31].

Weighted training is used to address the problem of class imbalance in classification. The typical proportion of positive and negative data is approximately 1 and 99%, respectively. This class imbalance could severely affect the performance of the classification model, thereby making the model biased towards the negative class, and thus incapable of identifying the samples of the positive class. Weighted training deletes this bias by giving larger weights to the positive class. For weighted training, first, the weights are estimated based on the proportion of available instances in each class. Then, these weights are used during training to adjust the loss function that offers better performance by reducing bias as well as generalization over all classes.

The performance of the models was assessed using metrics like precision, recall and F1 score [32]:


$$\begin{array}{c} Precision=\frac{True\;Positives\;(TP)}{True\;Positives\;(TP)+False\;Positives\;(FP)} \end{array}$$ (1)

$$\begin{array}{c} Recall=\frac{True\;Positives\;(TP)}{True\;Positives\;(TP)+False\;Negatives\;(FN)} \end{array}$$ (2)

$$\begin{array}{c} F\text{1}\;score=\frac{\text{2}\text{ x }Precision\text{ x }Recall}{Precision+Recall} \end{array}$$ (3)

To improve the precision, a threshold to the classification probability was introduced [33]. This threshold removes events from the classification where the probability calculated by the model falls below a certain value. While a fraction of events remains unclassified, those that are classified have a higher classification accuracy. Events to which the model assigns a higher confidence have an increased likelihood of being correctly classified.

## 3. Results

### 3.1. Comparison of light scattering properties of sporozoites and residual mosquito debris using MAPS-FC

To evaluate whether *P. falciparum* sporozoites can be distinguished from contaminating mosquito cells and debris without relying on fluorescent labelling, we applied multi-angle pulse shape flow cytometry (MAPS-FC). This technique records the full light scattering pulse shape during particle transit through the laser focus at multiple scattering angles, offering richer information than conventional scatter parameters. The fluorescence signal of the GFP-expressing *Pf*OBC13^GFP^ sporozoites [20] was used for validation purposes. Although a few reports on distinguishing *Plasmodium* sporozoites from host cells using conventional flow cytometry exist [21,22], this task appears challenging (see also Supporting Information Section 2 in S1 File), particularly when the events of interest represent a small fraction of the total population.

We analyzed samples from five biological replicates obtained on five different days of measurement, containing both sporozoites and residual mosquito cells. The GFP fluorescence signal was used for gating the sporozoite events, revealing that they accounted for less than 1% of all events (Figure S2 in S1 File). Figure 3(a) shows the scattered light intensity distribution in two channels of MAPS-FC which resemble FSC and SSC of a conventional flow cytometer. In these parameters, GFP-positive sporozoites were not separable from the broader GFP-negative background population. Fig 3(b-d) shows MAPS-FC pulse shape data captured at three different detection angles. Pulses of mosquito cells (GFP negative) from three different regions indicated in panel (a) are shown. Pulses in regions 1 and 3 clearly distinguish themselves from the sporozoites due to their much lower or higher amplitude. Therefore, only the averages are displayed. Pulses from region 2 have similar pulse amplitudes. However, from both the average and the individual pulses one can see that they differ in shape from the pulses of the sporozoites. The pulse shapes reveal characteristic features that may enable discrimination of sporozoites (GFP positive) from non-sporozoite events (GFP negative). Of note, these pulse shapes vary with scattering angle and exhibit heterogeneity within the GFP-positive population. The scattered light pulses in the integrating SSC are similar in structure between events with an extended plateau region and short rising and trailing edges. In contrast, SSC45 detects high intensity around the time points where a sporozoite enters and leaves the laser spot while the intensity is low when the sporozoite is in the middle of the spot. The FSC23 pulses show a Gaussian-like shape with continuous rising and falling phases and intensity peaking when the sporozoite is centered in the beam. Sporozoites are elongated in shape. Due to the acceleration during hydrodynamic focusing, they will orient along their long axis [34,35]. Tumbling due to a transverse velocity gradient is only expected further upstream than the point of analysis [34]. Thus, the vast majority of sporozoites is oriented along the flow direction when they pass the laser focus.

**Fig 3:**
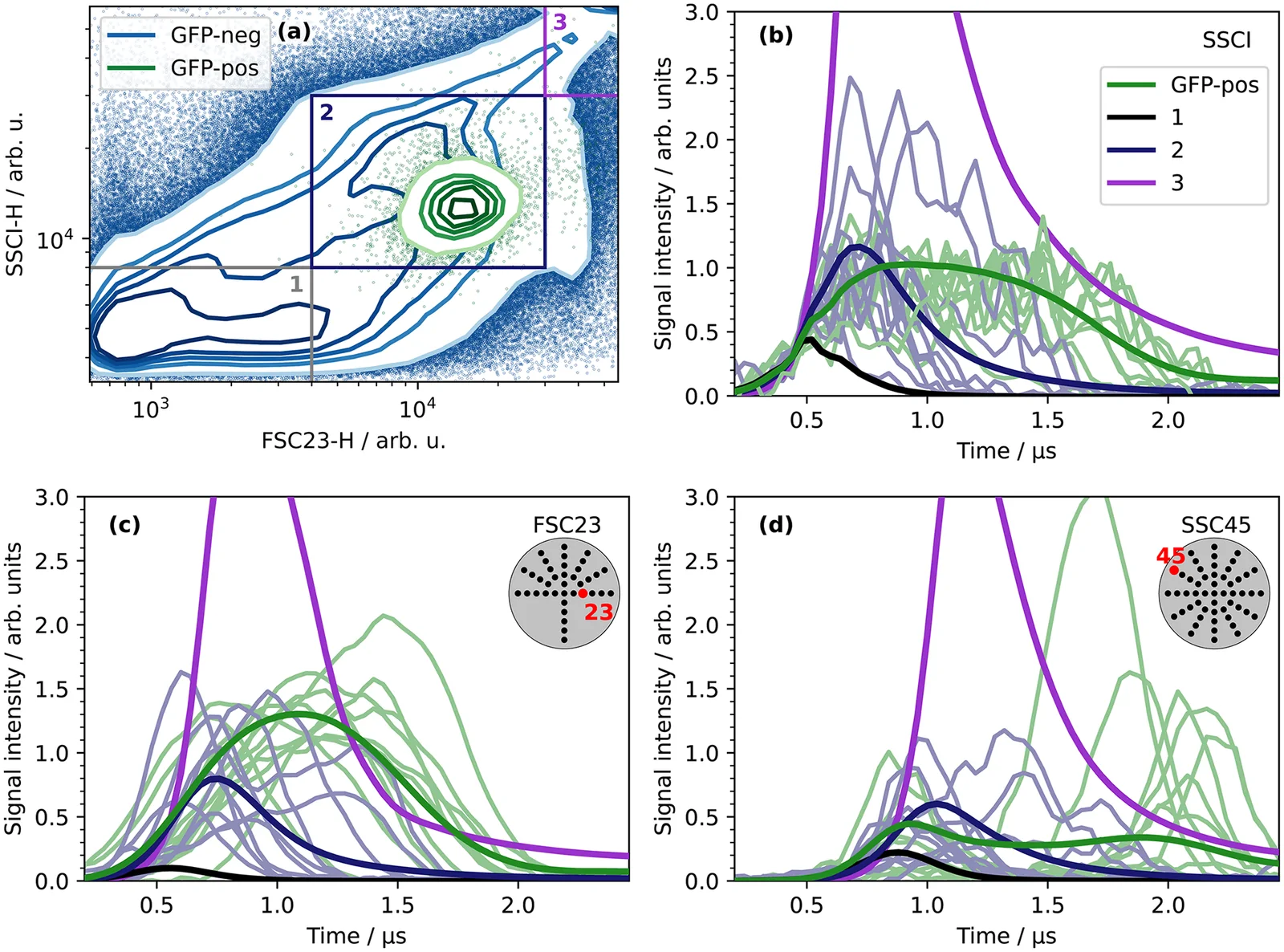
Comparison of the light scattering properties of sporozoites and residual mosquito debris. (a) Overlay of light scattering intensity in two channels resembling conventional FSC/SSC plots. The regions marked 1-3 are used for studying individual or average pulses. (b-d) Averages (solid lines) and exemplary (dim lines, only region 2 and GFP-positive events) pulse shapes of GFP-positive sporozoites and other GFP-negative particles from the three regions indicated in panel (a). The gating for GFP-positive events is given in Figure S2 in S1 File. The panels represent different detection channels (FSC and SSC angles). The insets show the fiber positions, i.e., the corresponding scattering angles. SSCI integrates over a wider angular range. In addition to the channels shown, four further scatter channels (FSC15, FSC19, SSC35 and SSC45) were included in the analysis. Altogether, seven light-scattering channels (SSCI, SSC27, SSC35, SSC45, FSC15, FSC19 and FSC23) were used for feature extraction and model training.

### 3.2. AI-based classification of sporozoite pulses using single-dataset training

In order to distinguish sporozoites from mosquito debris, the pulse shapes were classified using the AI model trained on the ground truth defined by the GFP fluorescence. In this analysis, the model was trained on a single dataset and tested with the remaining datasets, iterating the process on all the datasets. This approach aims to evaluate the model’s ability to generalize when trained on a single dataset and tested on the remaining ones. Each dataset from A-E consists of one million events, with positive-class events making up about 1.29% of dataset A, 0.88% of B, 0.73% of C, 0.37% of D, and 0.40% of E. The performance of the model was evaluated using precision, recall, and F1 scores as displayed in Fig 4. For each of the training datasets (A-E) labelled on the *x*-axis, the corresponding bars represent the performance on the remaining datasets, while the stars represent the model’s performance on the test split of the same dataset.

**Fig 4:**
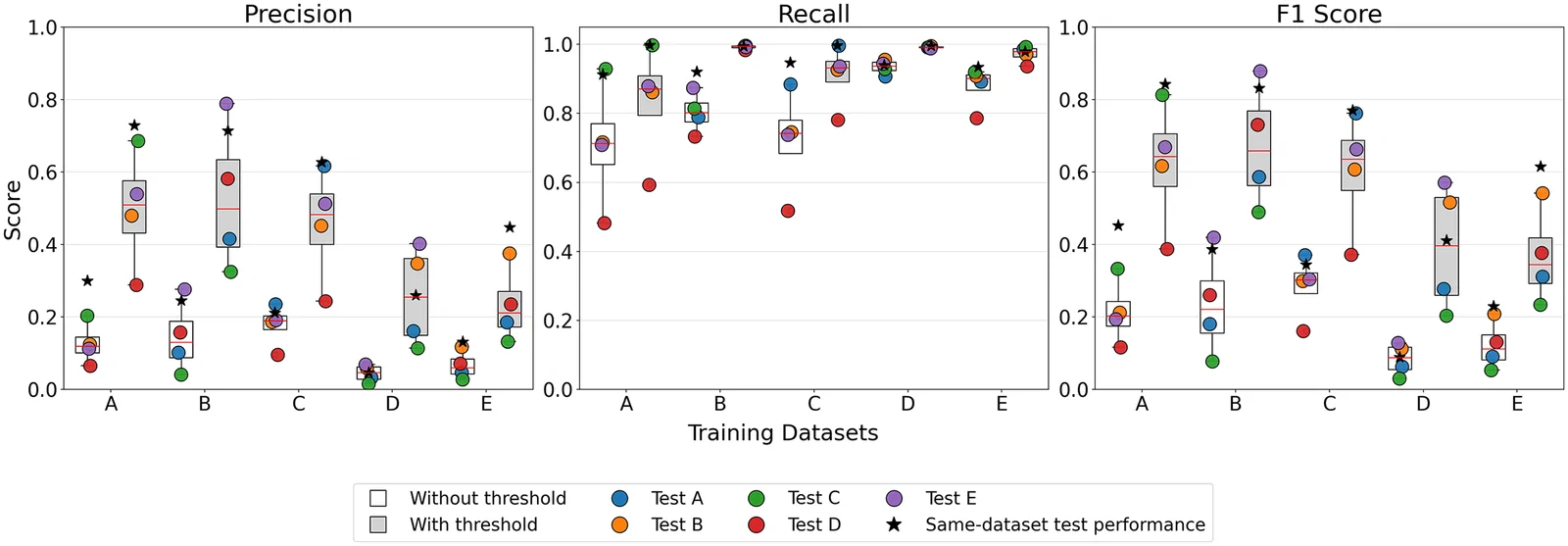
Precision, recall and F1 score of classifying parasites from the mosquito debris using different datasets. The x-axis shows the data over which the network was trained. The y-axis shows the performance of the model on different testing datasets, represented by colored dots for cross-dataset evaluation and the stars for the same dataset test performance.

Precision reflects the models ability of correctly identifying sporozoites without misclassifying non-sporozoites. With the highly imbalanced datasets, even a small absolute number of false positives can cause significant reduction in precision. Therefore, precision varies significantly across different training datasets, highlighting the dependency of decision boundaries on the specific characteristics of the training data. Models trained on datasets A, B and C resulted in relatively better precision in terms of median values. However, for models trained on D and E, the precision was consistently low for all test sets. Inter-dataset relationships were also observed: training on A gave the best precision on C and vice versa, indicating a similarity in the pulse shape distributions between the two datasets. Similarly, training on B and D resulted in best precision on E, and training on E produced best precision on B. These patterns suggest that the datasets segregate into two groups- {A, C} and {B, D, E}. Further, applying a 98% threshold on the model’s confidence score improved precision for a majority of the cases, though this involved exclusion of a substantial fraction of events from the analysis. On an average around 55% for the positive class and 52% for the negative class events were excluded depending on the training–testing combination (Figure S5 in S1 File). Using same-day datasets for testing (indicated by stars) gave better performance compared to most cross-dataset testing, which indicates the presence of diversity between different measurement days.

Recall measures the model’s ability to detect all the sporozoites, minimizing the false negatives. Across the different training datasets, recall stays consistently high ranging between 0.8 and 0.9. And when the model is tested on the same dataset, the recall reaches 1.0. This indicates that the model is capable of detecting sporozoites regardless of which dataset it was trained on.

The F1 score is defined as the harmonic mean of precision and recall which provides a balanced measure of overall classification performance. F1 scores remain moderate to low across most of the cross-dataset combinations driven by the small precision scores discussed above. Same test dataset (indicated by stars) performances consistently exceed cross-dataset results across all training datasets. This indicates that a model trained on a single dataset learns features that are dependent on the individual measurement and will include experimental noise, instrument variability and biological heterogeneity that are not representative of the full data space.

### 3.3. The effect of multi-dataset training on classification performance

To address the generalization limitation of single-dataset training, data measured on three different days (datasets A, B and D) were merged and used to train the model. This approach uses larger and diverse data for training which helps to capture a wider range of temporal variations in the pulse shapes and thus increases the model’s robustness. The remaining two datasets (C and E) were used as testing data. Each of the test sets contains approximately one million events of which <1% are sporozoites. Prior to classification, events exhibiting clipping of their pulse shapes (i.e., off-scale events resulting from detector saturation) were filtered out and excluded from the analysis. To improve classification precision, a 98% confidence threshold was applied to the model’s predictions, filtering out low-confidence events to reduce false positives while maintaining high true positive rate.

Fig 5 shows the scattered light intensity distributions obtained from SSCI and FSC23 channels to compare the ground truth labeling and the model’s predictions for distinguishing sporozoites (P) from non-sporozoites (NP) in dataset C. The dataset initially comprised 7,135 positive and 934,311 negative events, totaling 941,446. Without confidence thresholding, the predicted parasite events are spread across the feature space as in Fig 5(b). This leads to significant overlap with the non-sporozoite events resulting in a high number of false positives. This overlap reduces when the confidence threshold of 98% is applied to the model, see Fig 5(d). The retained set narrows to 5,650 positive and 223,484 negative events, totaling 229,134. Before thresholding, predictions correctly classified 887,743 of 941,446 events, an overall accuracy of 94.30%. It correctly identified 95.83% of true parasite events (6,837 of 7,135), but precision remained low at 11.35% due to 53,405 non-parasite events being misclassified as parasites. After thresholding, correct classifications rose to 225,401 of 229,134 events, an accuracy of 98.37%. Parasite detection improved to 99.88% (5,643 of 5,650), and precision increased more than fivefold to 60.23%, as misclassified non-parasite events dropped to 3,726. A similar pattern was observed using dataset E (see Supplementary Information Figure S4 in S1 File).

**Fig 5:**
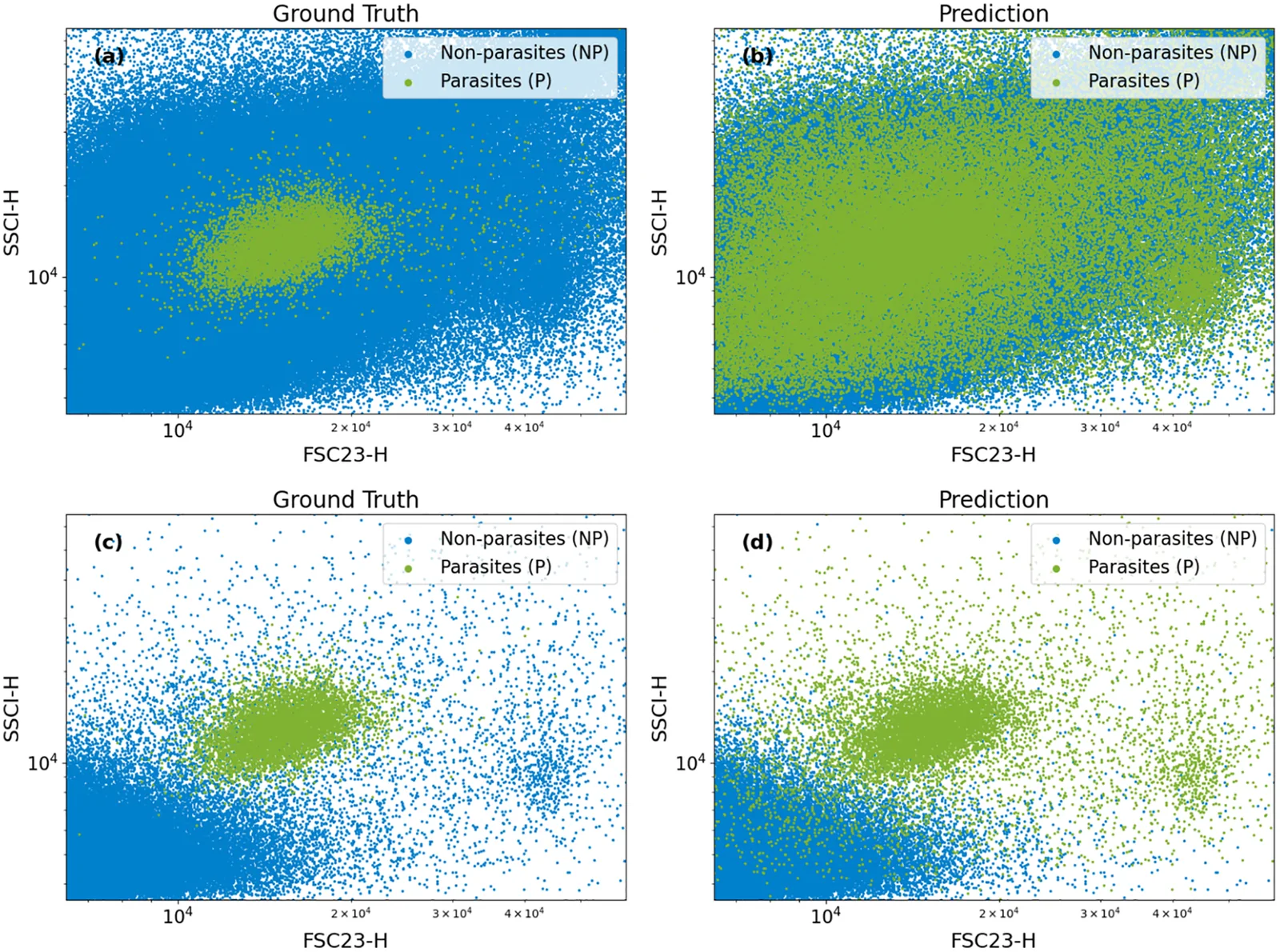
Scatter plots of peak pulse heights from dataset C using SSCI and FSC23. It shows the ground truth and predictions (a, b) without thresholding, and (c, d) with 98% thresholds.

However, applying thresholds introduces the risk of losing true positives with lower confidence scores. This trade-off between the sensitivity and specificity is demonstrated using Fig 6 which illustrates the impact of applying a threshold on the model classification accuracy in terms of precision and recall.

**Fig 6:**
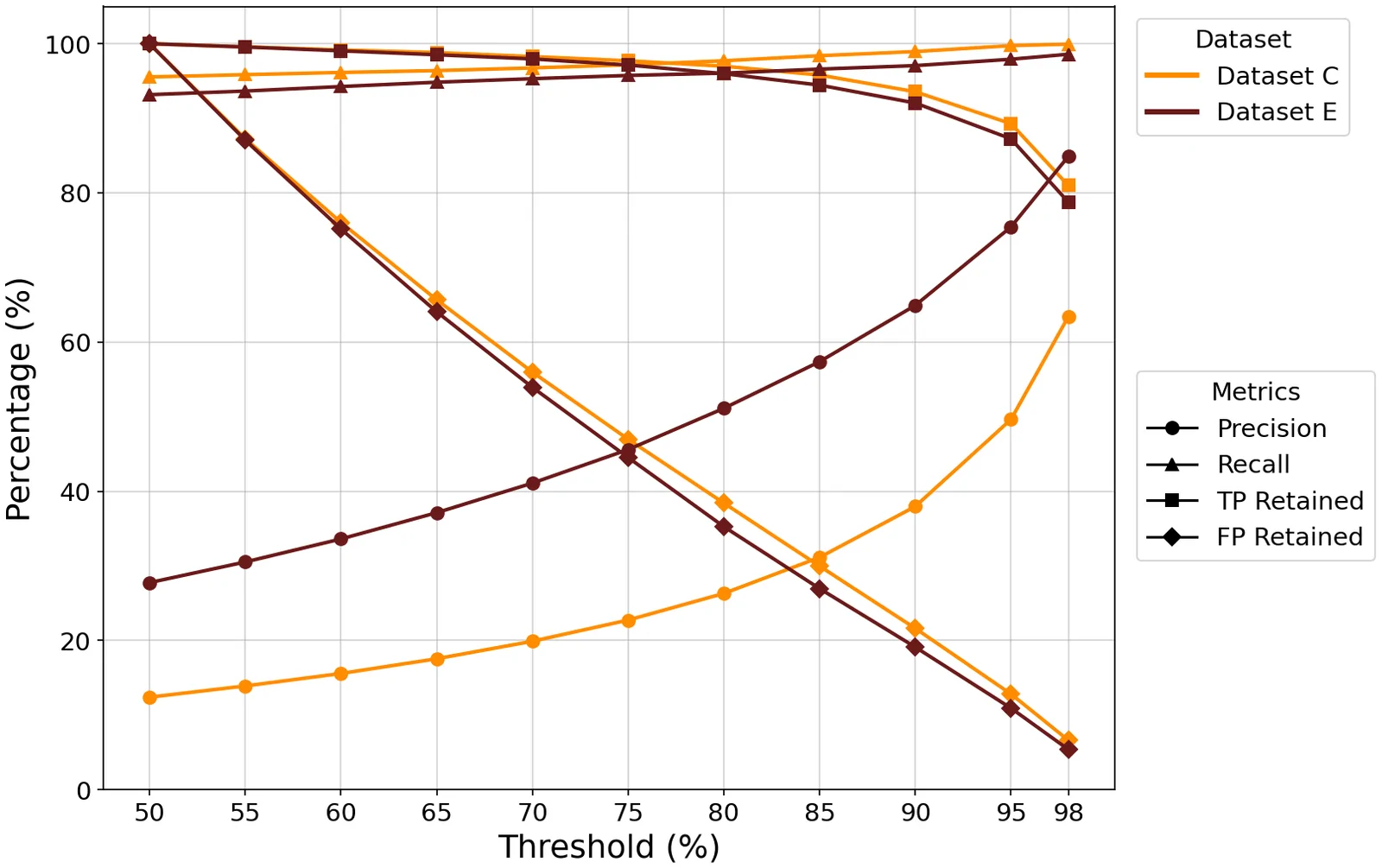
Performance metrics for test datasets C and E at different threshold percentages.

Without any threshold, the model overestimates the parasite class. This is evident from the higher number of false positives which in turn results in a lower precision score. When the thresholds are applied, there is an improvement in the accuracy – the number of false positives reduces significantly, while there is only a slight reduction in the number of true positives. The recall remains high in all the conditions with minimal variation. These results are consistent for both test datasets. Hence, confidence-based thresholding is an effective method to increase accuracy and reliability in parasite classification and effectively balances sensitivity and specificity. These results demonstrate that the model performance improves when trained on larger and more diverse dataset to generalize and predict more accurately on unseen data.

### 3.4. Characteristics of correctly and incorrectly classified pulse shapes

To better understand how the AI model distinguishes sporozoites from non-sporozoite events, we analyzed correctly classified and misclassified light scattering pulse shapes. To that end, we used the merged dataset for training and dataset C for testing with the threshold of 98%. This approach helps to reveal which pulse features the model relies on, and whether misclassifications may reflect limitations in the model or issues with the ground truth labelling. Fig 7 shows pulse shapes, where the bold curves represent mean pulse shapes and the lighter curves depict individual events. Correctly classified sporozoite (green) and non-sporozoite (black) events exhibit clearly distinct and well-separated temporal profiles. In contrast, misclassified events show more ambiguous pulse patterns. False positives (red) exhibit irregular intensity spikes and longer pulses with multiple peaks, closely mimicking the sporozoite profile. Notably, these false positives are largely confined to a distinct cluster which is characterized by increased FSC23 intensity in Fig 5(b) and (d). Though these events are GFP-negative according to the ground truth, visual inspection of the pulse shapes in this cluster shows that they are highly similar to those of GFP-positive sporozoites. This suggests that these events are morphologically parasite-like but lack GFP expression, accounting for approximately 9% of the total predicted parasites. False negatives (blue), in turn, tend to have narrower pulse shapes resembling those of true non-sporozoite events. Overall, this shows that the model strongly relies on the amplitude and the temporal features of the pulse for the classification.

**Fig 7:**
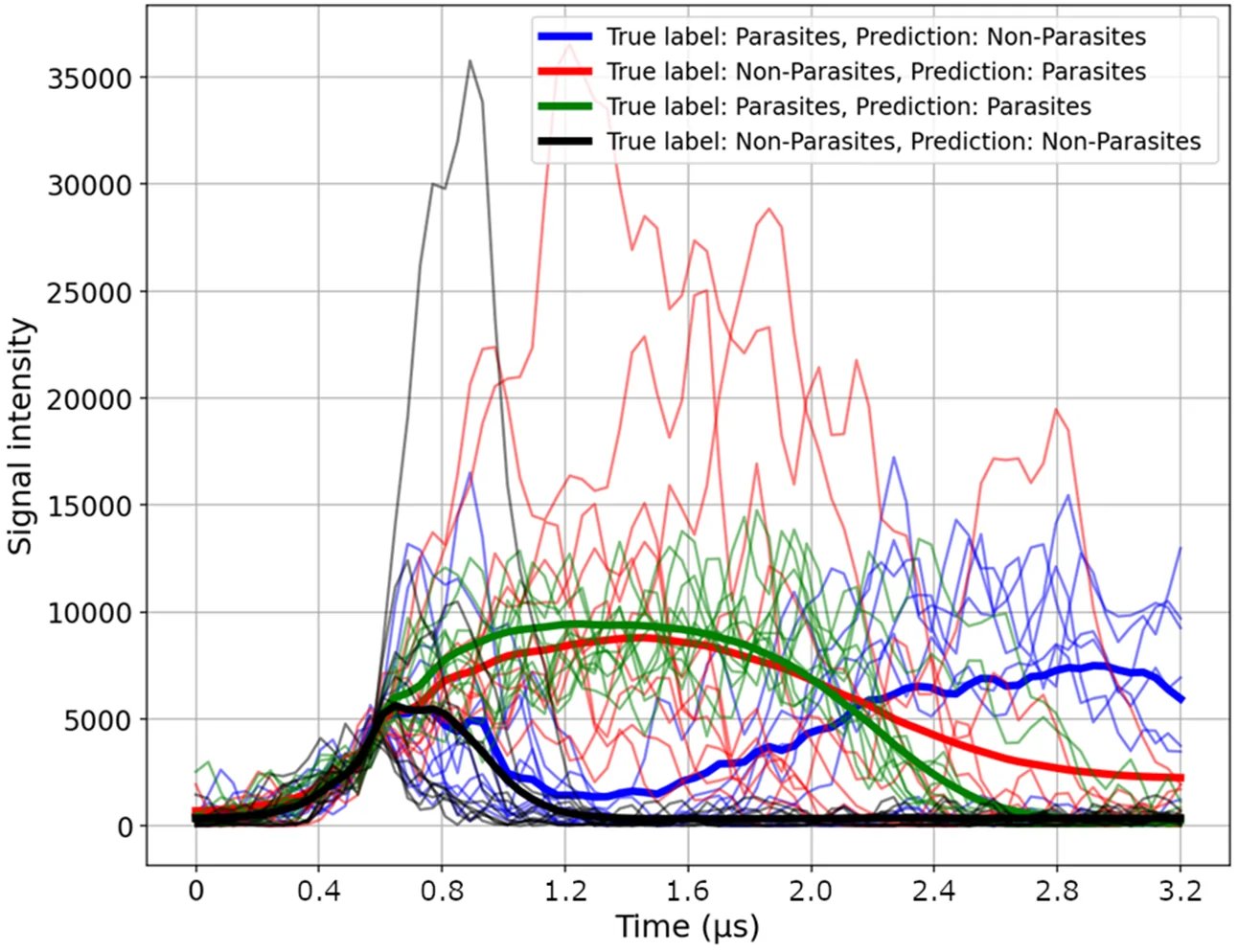
Light scattering pulse shapes from SSCI channel. These pulse shapes illustrate correct and incorrect classification of parasites and non-parasites for test set C. Broader curves represent the average pulse shapes, while the dimmer curves represent a subset of individual pulse shape.

## 4. Discussion

Using angle-resolved pulse shapes of scattered light in combination with an LSTM–based neural network, we demonstrated that *Plasmodium falciparum* sporozoites can be distinguished from *A. coluzzii* cells without further labelling. Data was acquired from multiple samples over the course of two months. Thus, the reproducibility of the approach could be validated.

Models trained on single datasets showed varying classification performance on unseen data, as reflected in differences in precision and recall. This could be caused by instrumental drift or differences in sample properties. Measurements performed with conventional flow cytometers on the same samples show qualitative variations in the scattered light distribution and shifts up to 20% in median intensity of the residual mosquito debris across the samples (data not shown). Considering that only approximately 1% of the total detected events are sporozoites, the datasets are strongly imbalanced, and classification purity (precision) is very sensitive to false positives. Another effect on performance was the similarity observed between datasets. Datasets A and C showed high mutual cross-dataset performance, implying high similarity between these datasets. Similarly, datasets B, D, and E showed high consistency in cross-dataset performance, implying similarity in these datasets. This might indicate the presence of similarity in the distributions of pulse shapes. Overall, performance was higher in same-dataset scenarios than in cross-dataset scenarios, validating the observation that single-dataset training accounts for variability within the training dataset but does not capture the heterogeneity present across different datasets.

To generalize our training model to include diversity, single datasets from multiple days were merged. Training the model on pulse shapes from a broader range of experimental conditions encompassed by a merged dataset improves generalization. Confidence thresholding also improves performance, precision in particular. This means that the threshold reduces the number of false positives more effectively than the number of true positives. Thus, the decisions of the models on true positives are more certain than those on the false positives. However, it comes at the cost of more unclassified events. When training with merged datasets, also the recall improves. Altogether, confidence-based thresholding is an effective method to increase accuracy and reliability in sporozoite classification, especially when using merged training datasets.

However, even with thresholding, some false positives still exist as a distinct population shifted to higher FSC23 intensities than the actual GFP-positive population (see Fig 5). These events might be related to sporozoites that have lost their integrity and thus lost or strongly reduced their GFP expression. Despite the slightly higher FSC23 peak intensity, SSCI pulse shapes (see Fig 7) of false positives are rather similar to those of true positive sporozoites. Consequently, a substantial fraction of the false positives likely arises from this GFP-negative, parasite-like population and might reflect limitations in defining the ground truth rather than purely erroneous model predictions.

To further validate the identity of the GFP-positive population used for ground-truth definition, we analyzed salivary gland samples from uninfected and infected mosquitoes (data not shown). A GFP-positive population was only observed in samples from infected mosquitoes, but not in uninfected mosquitoes. Furthermore, the GFP-positive population was also positive for anti-CSP antibodies [20], which is a well-established sporozoite surface marker.

Training on merged datasets also reduced the number of false negatives. False negatives (see Fig 7) tend to have narrower pulse shapes, e.g., in SSCI, resembling those of true non-sporozoite events. This suggests that the model strongly relies on the amplitude and the temporal features of the pulse for the classification. On the one hand, this could mean that the model indeed becomes better at recognizing the sporozoites. On the other hand, accounting for the possible presence of GFP-positive debris or dead sporozoites, although dead sporozoites were assumed to have lost fluorescence [36], the more diverse training dataset may have enabled improved recognition of such debris as sporozoites. The latter would be desirable from a purely algorithm-centered view but actually detrimental for a practical application. In short, the more generalized model recognizes sporozoites more consistently across datasets and it reduces the number of lost GFP-positive events as false negatives but it introduces more mosquito debris that were falsely classified as sporozoites. This emphasizes the dual challenge in classification tasks: enhancing the model’s ability to detect subtle features of the pulse shapes and maintain consistent ground truth to ensure reliable training. The trade-off could be mitigated by expanding the training set or by adding additional detectors that better distinguish the parasites from mosquito debris.

Generally, misclassifications need not be solely a function of model error, but also inconsistencies in the ground truth itself. Labelling by manual gating is prone to observer bias, overlapping of adjacent populations would lead to incorrect labelling of the events. Some of the events that were misclassified according to the ground truth, might actually be out-of-distribution cases that were mislabeled during ground truth definition.

As an alternative to using fluorescent reporter lines, other researchers used the light scattering parameters FSC pulse length (FSC-W) against SSC integrated intensity (SSC-A) of conventional flow cytometers to detect *Plasmodium yoelii* sporozoites and PbPfCSP sporozoites, *P. berghei* sporozoites genetically modified to express PfCSP in place of the endogenous PbCSP [21,22]. These results show that intrinsic scattering properties have already been used as informative features for the characterization of sporozoites. However, the purity achieved using this approach has not been reported. When we applied a similar gating strategy to our samples, we observed sporozoite purities ranging from only 5–30%, depending on the initial sporozoite content (see Supporting Information Section 3 in S1 File). Given the size variability of sporozoites among *Plasmodium* species, with *P. yoelii* being notably larger [37], it is likely that the above-mentioned gating strategy (FSC-W against SSC-A) may not be applicable to *P. falciparum* sporozoites. Generally, since the sporozoite population typically appears as a shoulder adjacent to the main population of host cell debris, the achievable purity is expected to strongly depend on sample composition, decreasing as the sporozoite-to-debris ratio declines. In contrast, the achievable purity in identification of sporozoites using the proposed approach combining MAPS-FC and neural networks is higher since light scattering characteristics are captured in greater detail. In summary, the approach of using multi-angle pulse shape flow cytometry (MAPS-FC) combined with deep neural networks for distinction of *P. falciparum* sporozoites from mosquito debris has proven feasible. Once a respective AI model has been trained, it could readily be applied to wild-type sporozoites.

This technique expands the analytical potential of flow cytometry in malaria research and beyond. Although our current setup is capable of basic cell sorting [6], it cannot apply a neural network for this purpose, yet. Future development, however, is supposed to enable real-time, label-free sporozoite sorting exploiting artificial intelligence for sorting decisions at a throughput comparable to state-of-the-art droplet sorters. Employing a droplet sorter for sporozoite isolation takes approximately 1 h for 2 ∙ 10^5^ sporozoites. Such advancements would therefore support high-throughput studies of sporozoite biology and accelerate the development of next-generation malaria interventions.

## Supporting information

S1 File Supporting Information. All references to Supporting Information in the manuscript refer to the Supporting Information Document.
(PDF)
