# Supplementary material for: Identification of Plasmodium sporozoites using angle-resolved scattered light pulse shapes
Source: PLoS One. 2026 Nov 5;21(11):e0354060. doi: 10.1371/journal.pone.0354060 (PMC13739179; doi:10.1371/journal.pone.0354060)
Supplement: Supplementary file 1 [file pone.0354060.s001.pdf]

Supporting Information:
Identification of Plasmodium sporozoites using angle-resolved scattered light pulse shapes

Vinod Devaraj[1,*], Sophia Kahler[2,*], Daniel Kage[1,*], Elena A. Levashina[2], Toralf Kaiser[1]

1. German Rheumatology Research Center (DRFZ) - Flow Cytometry Core Facility, Charitéplatz 1 (Virchowweg 12), 10117 Berlin, Germany
2. Max-Planck-Institut für Infektionsbiologie, Charitéplatz 1 (Virchowweg 12), 10117 Berlin, Germany

* Authors contributed equally

S1. Optical setup

The schematic drawing in Figure S1 shows a top view on the optical setup.

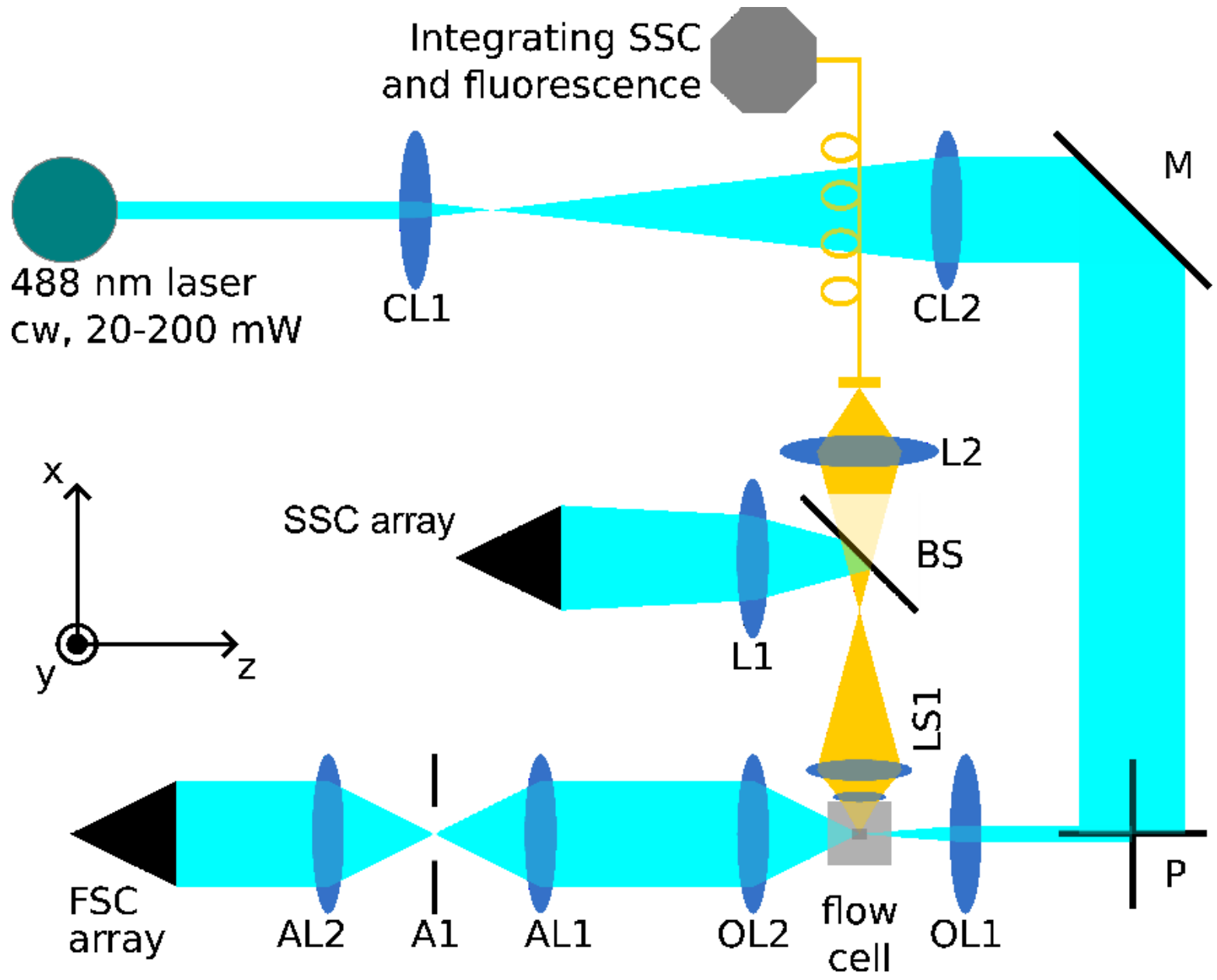

Figure S1: Top view on the optical layout of the custom flow cytometer.

The optical components of the setup are listed in Table S1.

CL1	cylindrical lens, *f*=50 mm	A1	50 µm slit along *x*
CL2	cylindrical lens, *f*=300 mm	LS1	BD fluorescence/SSC optics
M, P	metallic mirrors / periscope	BS	Dichroic beam splitter 488 nm
OL1 & 2	objective lenses, *f*=20 mm	L1	spherical lens, *f*=50 mm
AL1 & 2	achromatic compound lens, *f*=50 mm	L2	spherical lens, *f*=15 mm

Table S1: List of optical components.

S2. Gating

For defining the ground truth to train the algorithm, for cross-checking the classification, and other data analysis questions, the GFP-positive fraction of the sporozoites was gated according to the approach outlined in Figure S2.

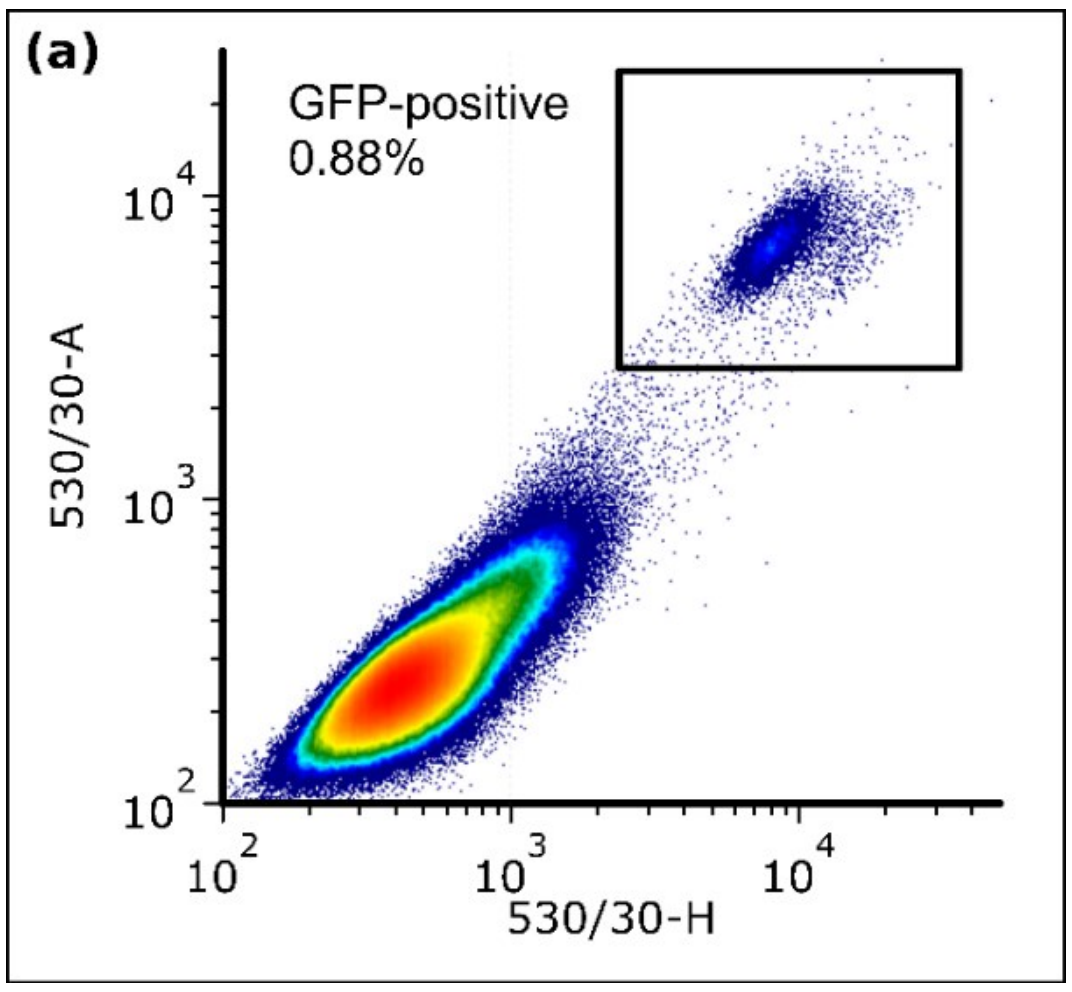

Figure S2: Gatting approach for sporozoites using the GFP fluorescence of the reporter line.

S3. Comparison of conventional scatter gating with MAPS-FC

Other research groups demonstrated the detection of *Plasmodium* sporozoites using conventional 'area' (A) and 'width' (W) parameters of scattered light[1,2]. We applied a comparable gating to our data as displayed in Figure S3(a). The obtained purity of this gating strategy can be deduced from Figure S3(b). In this case, a purity of around 30 % is obtained. The values for the other datasets vary between 5 and 30 %.

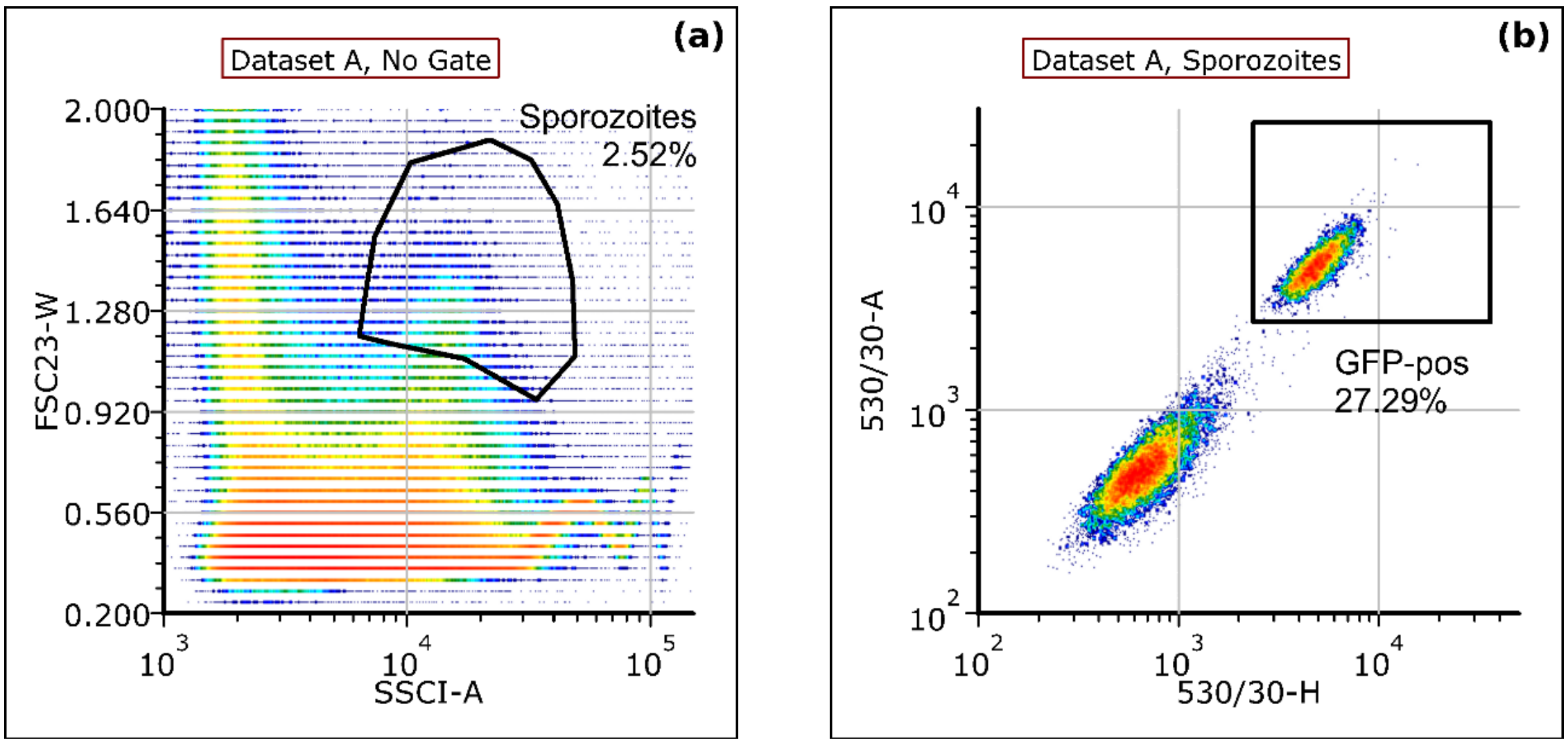

Figure S3: (a) Gating for Plasmodium sporozoites in conventional scattered light parameters. (b) Distribution of GFP-positive and -negative events in the gate from panel (a).

S4. Impact of thresholding on classification confidence

Similar to dataset B, in the absence of a confidence threshold, the predicted parasite events are distributed across the feature space and have a substantial overlap with the non-sporozoite events. This leads to increased number of false positives and consequently reduces the precision of identification. Applying a confidence threshold reduces the number of false positives, thereby improving prediction specificity while retaining dominant sporozoite population.

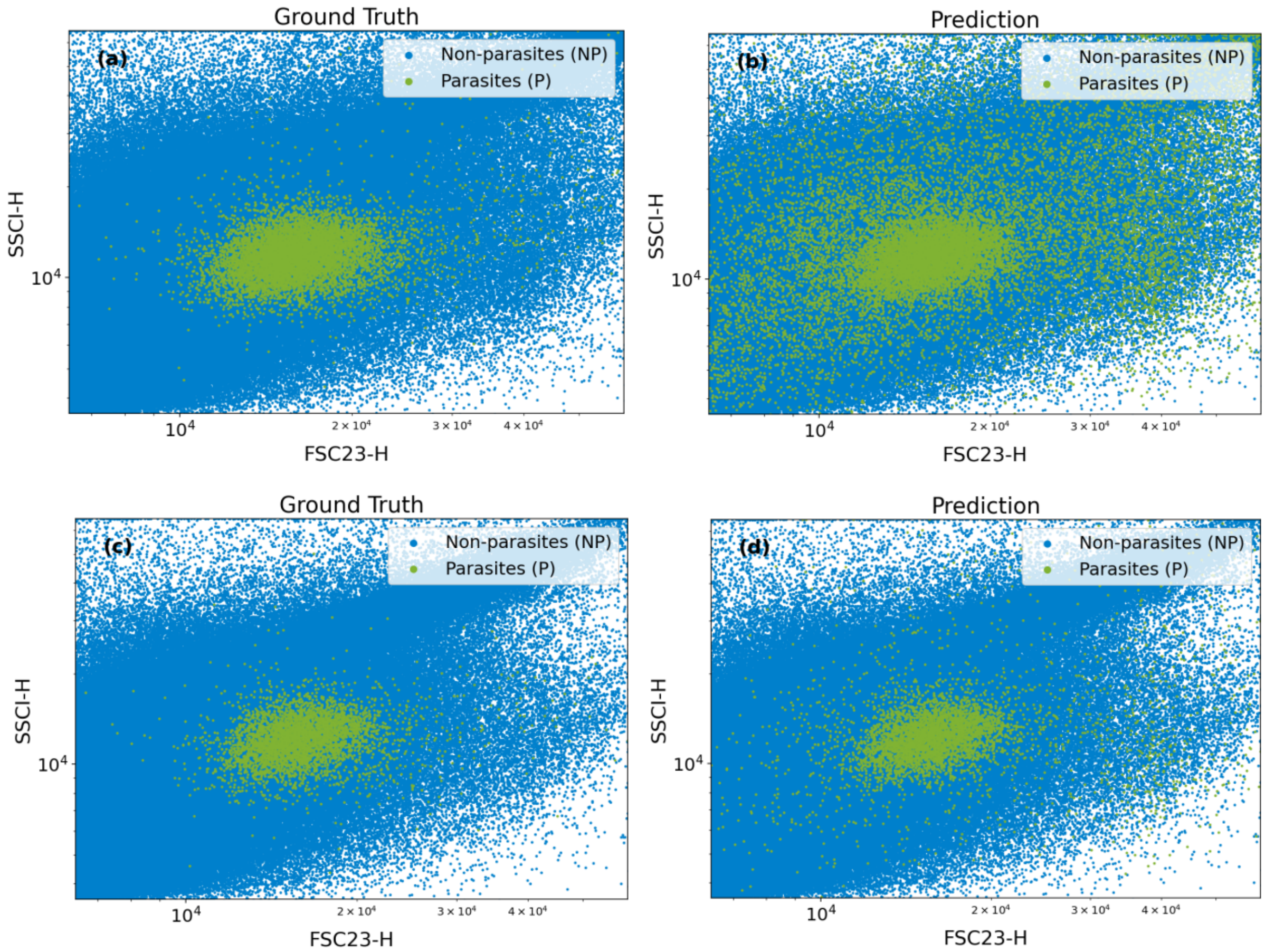

Figure S4: Scatter plots of peak pulse heights from dataset E using SSCI and FSC-23, showing the ground truth (a) and prediction (b) with threshold set at 95%.

S5. Event exclusion due to the confidence threshold

The confidence threshold was introduced to study its impact on the classification metrics. Using such a threshold increases the precision but leaves a fraction of events unclassified. This trade-off is beneficial for downstream applications such as sorting pure sporozoites. Further, the exclusion rate of true positive events decreases when training is performed on merged datasets rather than a single day, reflecting the improved generalization discussed in the manuscript.

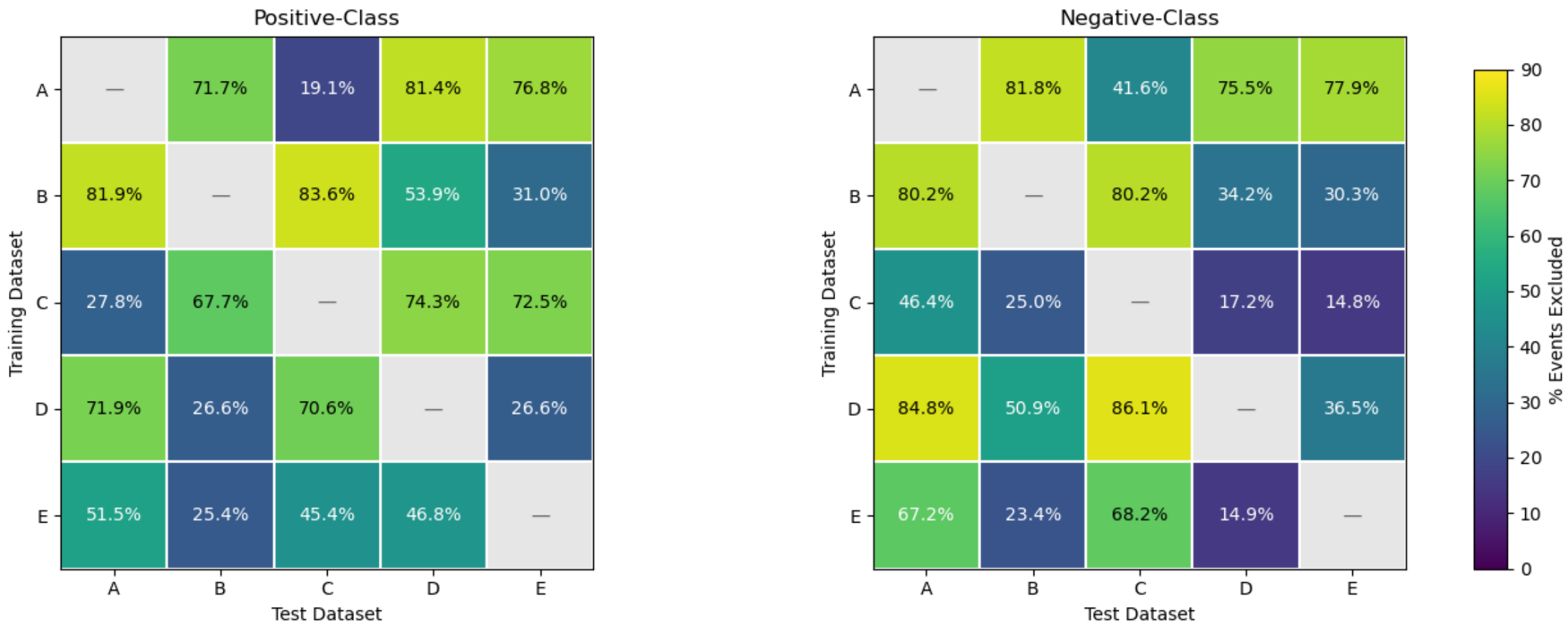

Figure S5: Number of events excluded when a 98% confidence threshold is applied. The left panel shows the effect on the sporozoite class, the right panel that on the mosquito host cell class. All combinations of training and testing sets are considered.
