# Supplementary material for: Identification of Plasmodium sporozoites using angle-resolved scattered light pulse shapes
Source: PLoS One. 2026 Nov 5;21(11):e0354060. doi: 10.1371/journal.pone.0354060 (PMC13739179; doi:10.1371/journal.pone.0354060)
Supplement: Supplementary file 2 [file pone.0354060.s003.docx]

**Response to Editor and Reviewer Comments**

We would like to thank the editor and reviewers for their comments which helped to improve the quality of the manuscript. We have addressed all the comments and suggestions with the responses below.

Response to Editor Comments

1. Although Devaraj et al. describe a novel methodology, multi-angle pulse shape flow cytometry, for sorting label-free Plasmodium sporozoites, the method still requires mosquito dissection. In addition, flow cytometric cell sorting can be relatively time-consuming and may be costly or inaccessible for some research laboratories. It would therefore be helpful if the authors could provide information on the time required to process and sort sporozoites from a typical experimental sample for example, salivary glands dissected from 50 mosquitoes and discuss the scalability of the method.

The method described in this manuscript is not limited to sporozoites but can be applied to many cell types and complex tissues. Here we use one example of how it can detect sporozoites within the mosquito cells and debris as we have a good test system based on transgenic sporozoites that constitutively express GFP as a control for specificity. Currently, there is no possibility to use the method for sporozoite sorting. The label-free sorting may be required for situations where no reporter-expressing cells are available or if we take an example of sporozoites, some clinical applications may need non-transgenic parasite material. While the requirement for mosquito dissection will persist, this method will enable sporozoite identification in the complex mixture and, potentially, sorting when the technology will be developed. In these experiments, dissection of 50 mosquitoes per experiment took us around 1 h, and the same time was required for FACS analysis.

2. In particular, can this approach be efficiently used to obtain large numbers of purified sporozoites for larger-scale experiments, such as in vitro infection of a large number of hepatocytes? This is an important practical consideration because sporozoites are generally used on the same day as extraction, and their infectivity progressively declines over time, even when maintained on ice. If sorting substantially increases the processing time for large samples, this could limit the practical utility of the method for experiments requiring high numbers of viable and infectious sporozoites.

The current study reported for the first time label-free identification of 1% of sporozoites in the heterogeneous mosquito samples. The potential application to sort the sporozoites has not been implemented yet. However, the timeframe for sporozoite identification by this method is compatible with the future sorting applications.

Response to Reviewer 1

1. It would be more impactful if they could show if the signal that they can detect is indeed the sporozoites. They could have used an imging cytometer (like an Imagestream) to show that the GFP signal is indeed coming from the sporozoites and that the populations that they are analysing are infact sporozoites and not debris. If the authors do not have access to such system, perhaps they could even do a simple negative staining from the isolated population to show sporozoites there and no sporozoites from the rest of the sample.

To address this question, we performed the following control experiment: salivary glands were dissected from both uninfected mosquitoes (negative control) and *Plasmodium*-infected mosquitoes, and the resulting samples were analyzed in a standard flow cytometer. As shown in the figure below, the negative control sample from uninfected mosquitoes (left panel) did not have GFP-positive population (circled area), whereas the sample from infected mosquitoes (right panel) features a clearly distinguishable GFP-positive cell population. This confirms that the specificity of the detected GFP signal. We have added a paragraph to the discussion section of the revised manuscript to reflect this point (line 373-377 in Revised Manuscript with Track Changes).

2. The authors say that they dissected 30 to 120 mosquitoes to get the sporozoites. But no mention on the total numbers of sporozoites per salivary glad or per mosquito in the samples analysed. This would help the readers better understand the size of the analysed population and the detection capabilities of this technique in comparison with other ones.
   The authors say that pulse shapes vary with scatering angle and exhibit heterogeneity within GFP-positive population (lines 193-195). But they do not dwell further on this matter in the discussion. It would be good if the authors could discuss this issue more. One small question - have the authors tried different fluorescence channels? Like mCherry or even 647nm range?

In our experiments, one mosquito contained between 20,000 to 60,000 sporozoites (average of ~34,000 sporozoites per mosquito). We dissected more mosquitoes than needed for this study to capture the natural heterogeneity of sporozoite populations across different mosquitoes and to perform other experiments on the same day. We have added information to the Methods Section of our manuscript (lines 97 – 99 in Revised Manuscript with Track Changes). We also extended the discussion of the pulse shapes of the sporozoites in lines 211-219 of the Revised Manuscript with Track Changes. Finally, the GFP fluorescence was specific to 488 nm excitation channel as observed by fluorescence microscopy.

Response to Reviewer 2

1. Can the method differentiate between midgut and salivary gland sporozoites?

In this study we dissected salivary gland sporozoites 14 and 15 days after infection. To enrich the samples for midgut sporozoites, dissections are usually done at earlier time points. Therefore, such differentiation is not possible with the set-up we used. Furthermore, there is no good marker to distinguish between the midgut and salivary gland sporozoites, which would complicate the validation of the sporozoite types if detected.

2. Can the method identify both from whole mosquito samples? (These could be performed by almost anyone).

Whole mosquito samples would drastically decrease the proportion of sporozoites relative to mosquito cells and result in much longer detection times.

3. How many events are classified false positive if uninfected mosquitoes are analyzed?

The false positive rate of uninfected mosquitoes was approximately 0.89%.

Minor comments:

1. Is there data from an Imaging Flow Cytometry system of sporozoites, i.e. is there any indication of the orientation relative to the flow direction? Briefly discuss

We agree with the reviewer that the orientation of the sporozoites would have an influence on the observed light scattering patterns. However, the hydrodynamic focusing aligns sporozoites along their long axis. Subsequent tumbling, and therefore deviation from this alignment, may occur in parabolic flow. In our system, the point of analysis is located before the parabolic flow has formed and therefore only minor deviations due to particle rotation are assumed. We added an explanation with literature references in our manuscript, lines 216-219 of the Revised Manuscript with Track Changes.

2. Figure 1: The figure is optically not appealing, and should be improved by adding some contrast, direction of light path and maybe a color code for light source and light detector. The design of the FSC and SSC should be explained in the text or figure text and separated in the figure from the main setup (maybe with A, B and C). Can you provide a real picture of the detector? Also, the positioning of the Y Z X and X Y Z coordinates is unclear what they relate to.

The authors acknowledge that the figure layout was in need of improvement. We separated the layouts of the fiber arrays from the schematic drawing of the setup, and added panel labels. The figure caption was extended to provide better explanations.

3. Figure 3: You show a wide array of signals for the GFP-neg cells in 3A which represent more than 99% of the events, but in B-D most events shown are GFP-positive. I would suggest to indicate areas in the blot 3A which are close to the GFP-pos sample (maybe 3 regions around the GFP-pos area) and 1 area closed to each corner of the blot, and then show at least 5 GFP-neg cells of all these 7 areas in 3B, 3C and 3D. If they are very similar in signal maybe the average signal can be shown in Figure 3, the individual blots should go into a file S3.
   Unrelated question: How is the Time 0 for 3B,C and D set? Relative to the FSC signal, to the peak or a certain signal over threshold?

The authors thank the reviewer for raising awareness that the diversity of pulses of mosquito host cells is not represented in this figure. We have overhauled Fig. 3 accordingly to now show three gates for mosquito host cells. From within these gates, pulses (average or individual examples) are displayed along with the sporozoite pulses. The Figure caption was adapted to explain the new features. Furthermore, descriptive text was added in lines 203-208.

The time point 0 is defined by the acquisition trigger. The trigger is a simple threshold trigger operating on the FSC23 channel. A respective explanation was added to the Methods Section in lines 129-131 of the Revised Manuscript with Track Changes.

4. Related question: Is it possible to generate a PCA analysis of your entire dataset? Does the temporal order of the individual SSC signals really matter? The time order question goes back to the question if sporozoites orient themself within the flow stream.

We thank the reviewer for this suggestion. We have generated a PCA analysis for the entire dataset (Datasets A–E), shown in the figure below. The plot illustrates the distribution of positive (sporozoite) and negative events along the first two principal components for each individual dataset, confirming a consistent overall separation between the two classes across all measurement days.

Regarding the second part of the question, we would like to clarify that PCA is not well-suited to address whether the temporal order of the SSC pulse signal is relevant to classification. PCA operates on the pulse data as a set of independent feature dimensions and identifies the directions of maximal variance across the dataset; it does not take into account the sequential relationship between time points within a single pulse. Consequently, PCA can reveal whether positive and negative events are separable based on their overall signal characteristics, but it cannot determine whether the order in which these characteristics occur along the pulse.

5. How many GFP positive and GFP negative events in training data sets A-E? What are the relative post infection days for these datasets? Are they from the same feed? Make a table. What are the relative infection rates and average salivary gland sporozoite numbers of each data set?

We thank the reviewer for raising this point and giving us the opportunity to clarify.

The fractions of GFP-positive events and the total number of events in all dataset are mentioned in lines 237-238 (Revised Manuscript with Track Changes) now.

Mosquito dissection was performed 14-15 days post infection for all datasets (cf. Methods Section 2.1.3). Every dataset stems from a different mosquito infection (feed). In our experiments, 90–100% of mosquitoes were infected, as evaluated by midgut oocyst counts. Salivary gland sporozoite numbers averaged 20,000–60,000 sporozoites per mosquito per experiment (overall average ~34,000 sporozoites per mosquito). We have added this information to the Methods section of the revised manuscript (lines 84-86 and 97-99 in Revised Manuscript with Track Changes).

6. Line 225: Explain the 98% threshold to the confidence score, this is unclear to me. Are all events with less than 98% of confidence just excluded from the analysis? If so, state the percentage of excluded events in all analysis in Figure 4.

We have clarified this in the manuscript, lines 181-185. The 98% confidence threshold refers to the probability the model assigns to its predicted class for each event. An event is only assigned a final label (positive or negative) if the model's confidence in that prediction is ≥98%; events falling below this threshold are excluded from the analysis as unclassified, rather than being forced into either class.

As requested, we have quantified the percentage of excluded events for every training/testing combination shown in Figure 4, separated by ground-truth class (Figure X in supporting information document). Exclusion rates vary considerably depending on the training–testing dataset pair, ranging from as low as 14.8–19.1% up to 81–86% for both positive and negative classes. In general, single-dataset training combined with testing on a different day's dataset excludes a large fraction of true positive events, since limited feature diversity from single-day training lowers the model's confidence on unseen days. This trade-off comes with a marked increase in precision/purity of the retained positive predictions, which is beneficial for downstream applications such as sorting pure sporozoites. Consistent with this, the exclusion rate of true positive events decreases when training is performed on merged, multi-day datasets rather than a single day, reflecting the improved generalization discussed in the manuscript.

7. Figure 4: The stars are not needed and overcomplicate the figure, just use the colors you already introduced.

The stars and the colored dots represent two conceptually different evaluations and cannot be merged into the existing color scheme without losing information. The colored dots show cross-dataset testing: the model is trained on one dataset (x-axis) and tested on each of the other four datasets, with color indicating the test dataset. The stars show a different condition: performance on a held-out test split from the *same* dataset used for training. This is the model's same-dataset baseline and needs to remain visually distinct from cross-dataset generalization performance. If we use a distinct color for this instead of a star, it would be indistinguishable from that dataset's cross-testing result, and the reader would lose the ability to tell same-dataset from cross-dataset performance. Therefore, we would retain the stars in the figure.

8. Figure 5: Indicate the numbers and percentage of overlap between prediction and ground truth for both A B and C D as well as the percentage of excluded events, separated into ground truth positive and negative events. (still keep Fig6).

We have added the requested quantitative details to the manuscript, lines 291-298, while retaining Figure 6 as suggested. The added details confirms that thresholding removes a substantially larger proportion of negative events than positive events, which is consistent with the observed improvement in precision.

9. Did you visually verify that 100% of salivary gland sporozoites are strong GFP positive using a microscope? Maybe the parasite-like population is not just parasite like but weak in GFP and under the threshold in FACS? Using a similar gating shown in Figure S2, sort the cells and perform live cell microscopy on these.

This has been verified in follow-up experiments from our group (Suárez-Cortés, manuscript in preparation), where in three independent experiments (N = 3, n = 200), all salivary gland sporozoites were confirmed to be GFP positive by live-cell fluorescence microscopy. In Figure S2, the GFP positive sporozoites form a distinct population with clear separation from the GFP negative background.

In addition, in a preprint from our group (Suárez-Cortés et al., 2023), it was shown that the GFP-positive population was also positive for anti-CSP antibodies, which is a well-established sporozoite surface marker and further confirms that the analyzed population indeed consists of sporozoites. We have added a paragraph to the discussion section of the revised manuscript to reflect this point (lines 373-377 in Revised Manuscript with Track Changes).
