# Supplementary material for: Identification of Plasmodium sporozoites using angle-resolved scattered light pulse shapes
Source: PLoS One. 2026 Nov 5;21(11):e0354060. doi: 10.1371/journal.pone.0354060 (PMC13739179; doi:10.1371/journal.pone.0354060)
Supplement: Peer review history

# Peer review: Decision Letter 0

**DOI:** 10.1371/journal.pone.0354060.r001

**Authors:** Shahin Tajeri

18 Aug 2026

*Plasmodium*

Dear Dr. Levashina,

Thank you for submitting your manuscript to PLOS ONE. After careful consideration, we feel that it has merit but does not fully meet PLOS ONE’s publication criteria as it currently stands. Therefore, we invite you to submit a revised version of the manuscript that addresses the points raised during the review process.

Please submit your revised manuscript by Sep 17 2026 11:59PM. If you will need more time than this to complete your revisions, please reply to this message or contact the journal office at plosone@plos.org. When you're ready to submit your revision, log on to https://www.editorialmanager.com/pone/ and select the 'Submissions Needing Revision' folder to locate your manuscript file.

- A letter that responds to each point raised by the academic editor and reviewer(s). You should upload this letter as a separate file labeled 'Response to Reviewers'.
- A marked-up copy of your manuscript that highlights changes made to the original version. You should upload this as a separate file labeled 'Revised Manuscript with Track Changes'.
- An unmarked version of your revised paper without tracked changes. You should upload this as a separate file labeled 'Manuscript'.

If you would like to make changes to your financial disclosure, please include your updated statement in your cover letter. Guidelines for resubmitting your figure files are available below the reviewer comments at the end of this letter.

If applicable, we recommend that you deposit your laboratory protocols in protocols.io to enhance the reproducibility of your results. Protocols.io assigns your protocol its own identifier (DOI) so that it can be cited independently in the future. For instructions see: https://journals.plos.org/plosone/s/submission-guidelines#loc-laboratory-protocols. Additionally, PLOS ONE offers an option for publishing peer-reviewed Lab Protocol articles, which describe protocols hosted on protocols.io. Read more information on sharing protocols at https://plos.org/protocols?utm_medium=editorial-email&utm_source=authorletters&utm_campaign=protocols.

As the corresponding author, your ORCID iD is verified in the submission system and will appear in the published article. PLOS supports the use of ORCID, and we encourage all coauthors to register for an ORCID iD and use it as well. Please encourage your coauthors to verify their ORCID iD within the submission system before final acceptance, as unverified ORCID iDs will not appear in the published article. *Only*  the individual author can complete the verification step; PLOS staff *cannot*  verify ORCID iDs on behalf of authors.

We look forward to receiving your revised manuscript.

Kind regards,

Shahin Tajeri, D.V.M. Ph.D.

Academic Editor

PLOS One

Journal Requirements:

When submitting your revision, we need you to address these additional requirements.

1. Please ensure that your manuscript meets PLOS ONE's style requirements, including those for file naming. The PLOS ONE style templates can be found at

https://journals.plos.org/plosone/s/file?id=wjVg/PLOSOne_formatting_sample_main_body.pdf and

https://journals.plos.org/plosone/s/file?id=ba62/PLOSOne_formatting_sample_title_authors_affiliations.pdf

2. Please note that PLOS One has specific guidelines on code sharing for submissions in which author-generated code underpins the findings in the manuscript. In these cases, we expect all author-generated code to be made available without restrictions upon publication of the work. Please review our guidelines at https://journals.plos.org/plosone/s/materials-and-software-sharing#loc-sharing-code and ensure that your code is shared in a way that follows best practice and facilitates reproducibility and reuse.

3. We note that the grant information you provided in the ‘Funding Information’ and ‘Financial Disclosure’ sections do not match.

When you resubmit, please ensure that you provide the correct grant numbers for the awards you received for your study in the ‘Funding Information’ section.

4. Thank you for stating the following financial disclosure: [VD and DK acknowledge funding by the German Research Foundation (DFG, grant number 491069896). SK was supported by the IMPRS fellowship].

Please state what role the funders took in the study.  If the funders had no role, please state: ""The funders had no role in study design, data collection and analysis, decision to publish, or preparation of the manuscript.""

If this statement is not correct you must amend it as needed.

Please include this amended Role of Funder statement in your cover letter; we will change the online submission form on your behalf.

5. Thank you for stating the following in the Acknowledgments Section of your manuscript: [VD and DK acknowledge funding by the German Research Foundation (DFG, grant number 491069896). SK was supported by the IMPRS fellowship. DK thanks the International Society for Advancement of Cytometry (ISAC) for continuous support. All authors thank the mosquito rearing and P. falciparum parasite culturing by Liane Spohr, Daniel Eyermann, Manuela Andres and thank Dr. Giulia Costa for her insightful scientific input. We also thank Dr. G. Costa and Dr. A. Wolf for proofreading the manuscript and fruitful discussions.]

We note that you have provided funding information that is not currently declared in your Funding Statement. However, funding information should not appear in the Acknowledgments section or other areas of your manuscript. We will only publish funding information present in the Funding Statement section of the online submission form.

Please remove any funding-related text from the manuscript and let us know how you would like to update your Funding Statement. Currently, your Funding Statement reads as follows: [VD and DK acknowledge funding by the German Research Foundation (DFG, grant number 491069896). SK was supported by the IMPRS fellowship].

Please include your amended statements within your cover letter; we will change the online submission form on your behalf.

6. We note that you have a patent relating to material pertinent to this article. Please provide an amended statement of Competing Interests to declare this patent (with details including name and number), along with any other relevant declarations relating to employment, consultancy, patents, products in development or modified products etc. Please confirm that this does not alter your adherence to all PLOS ONE policies on sharing data and materials, as detailed online in our guide for authors http://journals.plos.org/plosone/s/competing-interests by including the following statement: ""This does not alter our adherence to  PLOS ONE policies on sharing data and materials.” If there are restrictions on sharing of data and/or materials, please state these. Please note that we cannot proceed with consideration of your article until this information has been declared.

Please respond by return email and we will update the online submission form on your behalf.

7. Thank you for uploading your study's underlying data set. Unfortunately, the repository you have noted in your Data Availability statement does not qualify as an acceptable data repository according to PLOS's standards.

At this time, please upload the minimal data set necessary to replicate your study's findings to a stable, public repository (such as figshare or Dryad) and provide us with the relevant URLs, DOIs, or accession numbers that may be used to access these data. For a list of recommended repositories and additional information on PLOS standards for data deposition, please see https://journals.plos.org/plosone/s/recommended-repositories.

8. Please include captions for your Supporting Information files at the end of your manuscript, and update any in-text citations to match accordingly. Please see our Supporting Information guidelines for more information: http://journals.plos.org/plosone/s/supporting-information.

9. If the reviewer comments include a recommendation to cite specific previously published works, please review and evaluate these publications to determine whether they are relevant and should be cited. There is no requirement to cite these works unless the editor has indicated otherwise.

10. Please review your reference list to ensure that it is complete and correct. If you have cited papers that have been retracted, please include the rationale for doing so in the manuscript text, or remove these references and replace them with relevant current references. Any changes to the reference list should be mentioned in the rebuttal letter that accompanies your revised manuscript. If you need to cite a retracted article, indicate the article’s retracted status in the References list and also include a citation and full reference for the retraction notice.

Additional Editor Comments:

Although Devaraj et al. describe a novel methodology, multi-angle pulse shape flow cytometry, for sorting label-free Plasmodium sporozoites, the method still requires mosquito dissection. In addition, flow cytometric cell sorting can be relatively time-consuming and may be costly or inaccessible for some research laboratories. It would therefore be helpful if the authors could provide information on the time required to process and sort sporozoites from a typical experimental sample for example, salivary glands dissected from 50 mosquitoes and discuss the scalability of the method.

In particular, can this approach be efficiently used to obtain large numbers of purified sporozoites for larger-scale experiments, such as in vitro infection of a large number of hepatocytes? This is an important practical consideration because sporozoites are generally used on the same day as extraction, and their infectivity progressively declines over time, even when maintained on ice. If sorting substantially increases the processing time for large samples, this could limit the practical utility of the method for experiments requiring high numbers of viable and infectious sporozoites.

Reviewers' comments:

Reviewer's Responses to Questions

**Comments to the Author**

1. Is the manuscript technically sound, and do the data support the conclusions?

Reviewer #1: Yes

Reviewer #2: Yes

**********

2. Has the statistical analysis been performed appropriately and rigorously?

Reviewer #1: Yes

Reviewer #2: Yes

**********

3. Have the authors made all data underlying the findings in their manuscript fully available?

The PLOS Data policy

Reviewer #1: Yes

Reviewer #2: No

**********

4. Is the manuscript presented in an intelligible fashion and written in standard English?

Reviewer #1: Yes

Reviewer #2: Yes

**********

Reviewer #1: The manuscript from Devaraj et all. is a follow up from previous publications using a technology developed by some of the authors. In this work, the authors used a parasite - Plasmodium sporozoites - to see if their technology could be used to differentiate between small parasites and cellular debris (from mosquitoes salivary glands and other parts). The authors show some nice data on this. But it would be more impactful if they could show if the signal that they can detect is indeed the sporozoites. They could have used an imging cytometer (like an Imagestream) to show that the GFP signal is indeed coming from the sporozoites and that the populations that they are analysiing are infact sporozoites and not debris. If the authors do not have access to such system, perhaps they could even do a simple negative staining from the isolated population to show sporozoites there and no sporozoites from the rest of the sample.

The authors say that they dissected 30 to 120 mosquitoes to get the sporozoites. But no mention on the total numbers of sporozoites per salivary glad or per mosquito in the samples analysed. This would help the readers better understand the size of the analysed population and the detection capabilities of this technique in comparison with other ones.

The authors say that pulse shapes vary with scatering angle and exhibit heterogeneity within GFP-positive population (lines 193-195). But they do not dwell further on this matter in the discussion. It would be good if the authors could discuss this issue more. One small question - have the authors tried different fluorescence channels? Like mCherry or even 647nm range?

Reviewer #2: The authors describe a custom built FACs based system to identify Plasmodium sporozoites from scattered light with the help of deep learning-based analysis. With the help of multiple adjustments, they do reach a reasonable precision in their prediction which is measured using GFP expressing sporozoites. While this is great to show on its own, I am wondering on the potential use cases, except the suggested future use case of AI assisted cell sorting.

Sporozoite dissection from salivary glands is a difficult task itself, and the subsequent manual quantification via a Neubauer chamber is a far less difficult task, which precisely quantifies the amount of sporozoites with a near 100% precision if performed by a trained scientist. Sporozoite dissection varies greatly in purity depending on the skill level of the dissecting individual as well as the time used per mosquito. And in the presented study, dissection of the salivary gland is still required. What would be great to answer is:

1. Can the method differentiate between midgut and salivary gland sporozoites?

2. Can the method identify both from whole mosquito samples? (These could be performed by almost anyone).

3. How many events are classified false positive if uninfected mosquitoes are analyzed?

While the first 2 questions could be questions for follow up studies, the 3rd question should be adressed in this manuscript, along with the minor comments below:

minor comments:

Much of the analysis of the temporal pattern of scattered light to identify sporozoites relies on the assumption that all sporozoites will behave comparatively in the FACS. Sporozoites are among the most polarized cells and could potentially orient in all possible orientations within the FACS. Is there data from an Imaging Flow Cytometry system of sporozoites, i.e. is there any indication of the orientation relative to the flow direction? Briefly discuss

Figure 1: The figure is optically not appealing, and should be improved by adding some contrast, direction of light path and maybe a color code for light source and light detector. The design of the FSC and SSC should be explained in the text or figure text and separated in the figure from the main setup (maybe with A, B and C). Can you provide a real picture of the detector? Also, the positioning of the Y Z X and X Y Z coordinates is unclear what they relate to.

Figure 3:

You show a wide array of signals for the GFP-neg cells in 3A which represent more than 99% of the events, but in B-D most events shown are GFP-positive. I would suggest to indicate areas in the blot 3A which are close to the GFP-pos sample (maybe 3 regions around the GFP-pos area) and 1 area closed to each corner of the blot, and then show at least 5 GFP-neg cells of all these 7 areas in 3B, 3C and 3D. If they are very similar in signal maybe the average signal can be shown in Figure3, the individual blots should go into a file S3.

Unrelated question: How is the Time 0 for 3B,C and D set? Relative to the FSC signal, to the peak or a certain signal over threshold?

related question: Is it possible to generate a PCA analysis of your entire dataset? Does the temporal order of the individual SSC signals really matter? The time order quesion goes back to the question if sporozoites orient themself within the flow stream.

Training data sets: How many GFP positive and GFP negative events in training data sets A-E? What are the relative post infection days for these datasets? Are they from the same feed? Make a table. What are the relative infection rates and average salivary gland sporozoite numbers of each data set?

Line 225: Explain the 98% threshold to the confidence score, this is unclear to me. Are all events with less than 98% of confidence just excluded from the analysis? If so, state the percentage of excluded events in all analysis in Figure 4.

Figure 4: The stars are not needed and overcomplicate the figure, just use the colors you already introduced.

Figure 5: Indicate the numbers and percentage of overlap between prediction and ground truth for both A B and C D as well as the percentage of excluded events, separated into ground truth positive and negative events. (still keep Fig6).

Did you visually verify that 100% of salivary gland sporozoites are strong GFP positive using a microscope? Maybe the parasite-like population is not just parasite like but weak in GFP and under the threshold in FACS? Using a similar gating shown in Figure S2, sort the cells and perform live cell microscopy on these

**********

what does this mean?). If published, this will include your full peer review and any attached files.

If you choose “no”, your identity will remain anonymous but your review may still be made public.

**Do you want your identity to be public for this peer review?**  For information about this choice, including consent withdrawal, please see our Privacy Policy

Reviewer #1: No

Reviewer #2: No

**********

[NOTE: If reviewer comments were submitted as an attachment file, they will be attached to this email and accessible via the submission site. Please log into your account, locate the manuscript record, and check for the action link "View Attachments". If this link does not appear, there are no attachment files.]

To ensure your figures meet our technical requirements, please review our figure guidelines: https://journals.plos.org/plosone/s/figures

You may also use PLOS’s free figure tool, NAAS, to help you prepare publication quality figures: https://journals.plos.org/plosone/s/figures#loc-tools-for-figure-preparation.

NAAS will assess whether your figures meet our technical requirements by comparing each figure against our figure specifications.

# Author comment: Author response to Decision Letter 1

**DOI:** 10.1371/journal.pone.0354060.r002

17 Sep 2026

We would like to thank the editor and reviewers for their comments which helped to improve the quality of the manuscript. We have addressed all the comments and suggestions with the responses below.

Response to Editor Comments

1. Although Devaraj et al. describe a novel methodology, multi-angle pulse shape flow cytometry, for sorting label-free Plasmodium sporozoites, the method still requires mosquito dissection. In addition, flow cytometric cell sorting can be relatively time-consuming and may be costly or inaccessible for some research laboratories. It would therefore be helpful if the authors could provide information on the time required to process and sort sporozoites from a typical experimental sample for example, salivary glands dissected from 50 mosquitoes and discuss the scalability of the method.

The method described in this manuscript is not limited to sporozoites but can be applied to many cell types and complex tissues. Here we use one example of how it can detect sporozoites within the mosquito cells and debris as we have a good test system based on transgenic sporozoites that constitutively express GFP as a control for specificity. Currently, there is no possibility to use the method for sporozoite sorting. The label-free sorting may be required for situations where no reporter-expressing cells are available or if we take an example of sporozoites, some clinical applications may need non-transgenic parasite material. While the requirement for mosquito dissection will persist, this method will enable sporozoite identification in the complex mixture and, potentially, sorting when the technology will be developed. In these experiments, dissection of 50 mosquitoes per experiment took us around 1 h, and the same time was required for FACS analysis.

2. In particular, can this approach be efficiently used to obtain large numbers of purified sporozoites for larger-scale experiments, such as in vitro infection of a large number of hepatocytes? This is an important practical consideration because sporozoites are generally used on the same day as extraction, and their infectivity progressively declines over time, even when maintained on ice. If sorting substantially increases the processing time for large samples, this could limit the practical utility of the method for experiments requiring high numbers of viable and infectious sporozoites.

The current study reported for the first time label-free identification of 1% of sporozoites in the heterogeneous mosquito samples. The potential application to sort the sporozoites has not been implemented yet. However, the timeframe for sporozoite identification by this method is compatible with the future sorting applications.

Response to Reviewer 1

1. It would be more impactful if they could show if the signal that they can detect is indeed the sporozoites. They could have used an imging cytometer (like an Imagestream) to show that the GFP signal is indeed coming from the sporozoites and that the populations that they are analysing are infact sporozoites and not debris. If the authors do not have access to such system, perhaps they could even do a simple negative staining from the isolated population to show sporozoites there and no sporozoites from the rest of the sample.

To address this question, we performed the following control experiment: salivary glands were dissected from both uninfected mosquitoes (negative control) and Plasmodium-infected mosquitoes, and the resulting samples were analyzed in a standard flow cytometer. As shown in the figure below, the negative control sample from uninfected mosquitoes (left panel) did not have GFP-positive population (circled area), whereas the sample from infected mosquitoes (right panel) features a clearly distinguishable GFP-positive cell population. This confirms that the specificity of the detected GFP signal. We have added a paragraph to the discussion section of the revised manuscript to reflect this point (line 373-377 in Revised Manuscript with Track Changes).

2. The authors say that they dissected 30 to 120 mosquitoes to get the sporozoites. But no mention on the total numbers of sporozoites per salivary glad or per mosquito in the samples analysed. This would help the readers better understand the size of the analysed population and the detection capabilities of this technique in comparison with other ones.

The authors say that pulse shapes vary with scatering angle and exhibit heterogeneity within GFP-positive population (lines 193-195). But they do not dwell further on this matter in the discussion. It would be good if the authors could discuss this issue more. One small question - have the authors tried different fluorescence channels? Like mCherry or even 647nm range?

In our experiments, one mosquito contained between 20,000 to 60,000 sporozoites (average of ~34,000 sporozoites per mosquito). We dissected more mosquitoes than needed for this study to capture the natural heterogeneity of sporozoite populations across different mosquitoes and to perform other experiments on the same day. We have added information to the Methods Section of our manuscript (lines 97 – 99 in Revised Manuscript with Track Changes). We also extended the discussion of the pulse shapes of the sporozoites in lines 211-219 of the Revised Manuscript with Track Changes. Finally, the GFP fluorescence was specific to 488 nm excitation channel as observed by fluorescence microscopy.

Response to Reviewer 2

1. Can the method differentiate between midgut and salivary gland sporozoites?

In this study we dissected salivary gland sporozoites 14 and 15 days after infection. To enrich the samples for midgut sporozoites, dissections are usually done at earlier time points. Therefore, such differentiation is not possible with the set-up we used. Furthermore, there is no good marker to distinguish between the midgut and salivary gland sporozoites, which would complicate the validation of the sporozoite types if detected.

2. Can the method identify both from whole mosquito samples? (These could be performed by almost anyone).

Whole mosquito samples would drastically decrease the proportion of sporozoites relative to mosquito cells and result in much longer detection times.

3. How many events are classified false positive if uninfected mosquitoes are analyzed?

The false positive rate of uninfected mosquitoes was approximately 0.89%.

Minor comments:

1. Is there data from an Imaging Flow Cytometry system of sporozoites, i.e. is there any indication of the orientation relative to the flow direction? Briefly discuss

We agree with the reviewer that the orientation of the sporozoites would have an influence on the observed light scattering patterns. However, the hydrodynamic focusing aligns sporozoites along their long axis. Subsequent tumbling, and therefore deviation from this alignment, may occur in parabolic flow. In our system, the point of analysis is located before the parabolic flow has formed and therefore only minor deviations due to particle rotation are assumed. We added an explanation with literature references in our manuscript, lines 216-219 of the Revised Manuscript with Track Changes.

2. Figure 1: The figure is optically not appealing, and should be improved by adding some contrast, direction of light path and maybe a color code for light source and light detector. The design of the FSC and SSC should be explained in the text or figure text and separated in the figure from the main setup (maybe with A, B and C). Can you provide a real picture of the detector? Also, the positioning of the Y Z X and X Y Z coordinates is unclear what they relate to.

The authors acknowledge that the figure layout was in need of improvement. We separated the layouts of the fiber arrays from the schematic drawing of the setup, and added panel labels. The figure caption was extended to provide better explanations.

3. Figure 3: You show a wide array of signals for the GFP-neg cells in 3A which represent more than 99% of the events, but in B-D most events shown are GFP-positive. I would suggest to indicate areas in the blot 3A which are close to the GFP-pos sample (maybe 3 regions around the GFP-pos area) and 1 area closed to each corner of the blot, and then show at least 5 GFP-neg cells of all these 7 areas in 3B, 3C and 3D. If they are very similar in signal maybe the average signal can be shown in Figure 3, the individual blots should go into a file S3.

Unrelated question: How is the Time 0 for 3B,C and D set? Relative to the FSC signal, to the peak or a certain signal over threshold?

The authors thank the reviewer for raising awareness that the diversity of pulses of mosquito host cells is not represented in this figure. We have overhauled Fig. 3 accordingly to now show three gates for mosquito host cells. From within these gates, pulses (average or individual examples) are displayed along with the sporozoite pulses. The Figure caption was adapted to explain the new features. Furthermore, descriptive text was added in lines 203-208.

The time point 0 is defined by the acquisition trigger. The trigger is a simple threshold trigger operating on the FSC23 channel. A respective explanation was added to the Methods Section in lines 129-131 of the Revised Manuscript with Track Changes.

4. Related question: Is it possible to generate a PCA analysis of your entire dataset? Does the temporal order of the individual SSC signals really matter? The time order question goes back to the question if sporozoites orient themself within the flow stream.

We thank the reviewer for this suggestion. We have generated a PCA analysis for the entire dataset (Datasets A–E), shown in the figure below. The plot illustrates the distribution of positive (sporozoite) and negative events along the first two principal components for each individual dataset, confirming a consistent overall separation between the two classes across all measurement days.

Regarding the second part of the question, we would like to clarify that PCA is not well-suited to address whether the temporal order of the SSC pulse signal is relevant to classification. PCA operates on the pulse data as a set of independent feature dimensions and identifies the directions of maximal variance across the dataset; it does not take into account the sequential relationship between time points within a single pulse. Consequently, PCA can reveal whether positive and negative events are separable based on their overall signal characteristics, but it cannot determine whether the order in which these characteristics occur along the pulse.

5. How many GFP positive and GFP negative events in training data sets A-E? What are the relative post infection days for these datasets? Are they from the same feed? Make a table. What are the relative infection rates and average salivary gland sporozoite numbers of each data set?

We thank the reviewer for raising this point and giving us the opportunity to clarify.

The fractions of GFP-positive events and the total number of events in all dataset are mentioned in lines 237-238 (Revised Manuscript with Track Changes) now.

Mosquito dissection was performed 14-15 days post infection for all datasets (cf. Methods Section 2.1.3). Every dataset stems from a different mosquito infection (feed). In our experiments, 90–100% of mosquitoes were infected, as evaluated by midgut oocyst counts. Salivary gland sporozoite numbers averaged 20,000–60,000 sporozoites per mosquito per experiment (overall average ~34,000 sporozoites per mosquito). We have added this information to the Methods section of the revised manuscript (lines 84-86 and 97-99 in Revised Manuscript with Track Changes).

6. Line 225: Explain the 98% threshold to the confidence score, this is unclear to me. Are all events with less than 98% of confidence just excluded from the analysis? If so, state the percentage of excluded events in all analysis in Figure 4.

We have clarified this in the manuscript, lines 181-185. The 98% confidence threshold refers to the probability the model assigns to its predicted class for each event. An event is only assigned a final label (positive or negative) if the model's confidence in that prediction is ≥98%; events falling below this threshold are excluded from the analysis as unclassified, rather than being forced into either class.

As requested, we have quantified the percentage of excluded events for every training/testing combination shown in Figure 4, separated by ground-truth class (Figure X in supporting information document). Exclusion rates vary considerably depending on the training–testing dataset pair, ranging from as low as 14.8–19.1% up to 81–86% for both positive and negative classes. In general, single-dataset training combined with testing on a different day's dataset excludes a large fraction of true positive events, since limited feature diversity from single-day training lowers the model's confidence on unseen days. This trade-off comes with a marked increase in precision/purity of the retained positive predictions, which is beneficial for downstream applications such as sorting pure sporozoites. Consistent with this, the exclusion rate of true positive events decreases when training is performed on merged, multi-day datasets rather than a single day, reflecting the improved generalization discussed in the manuscript.

7. Figure 4: The stars are not needed and overcomplicate the figure, just use the colors you already introduced.

The stars and the colored dots represent two conceptually different evaluations and cannot be merged into the existing color scheme without losing information. The colored dots show cross-dataset testing: the model is trained on one dataset (x-axis) and tested on each of the other four datasets, with color indicating the test dataset. The stars show a different condition: performance on a held-out test split from the same dataset used for training. This is the model's same-dataset baseline and needs to remain visually distinct from cross-dataset generalization performance. If we use a distinct color for this instead of a star, it would be indistinguishable from that dataset's cross-testing result, and the reader would lose the ability to tell same-dataset from cross-dataset performance. Therefore, we would retain the stars in the figure.

8. Figure 5: Indicate the numbers and percentage of overlap between prediction and ground truth for both A B and C D as well as the percentage of excluded events, separated into ground truth positive and negative events. (still keep Fig6).

We have added the requested quantitative details to the manuscript, lines 291-298, while retaining Figure 6 as suggested. The added details confirms that thresholding removes a substantially larger proportion of negative events than positive events, which is consistent with the observed improvement in precision.

9. Did you visually verify that 100% of salivary gland sporozoites are strong GFP positive using a microscope? Maybe the parasite-like population is not just parasite like but weak in GFP and under the threshold in FACS? Using a similar gating shown in Figure S2, sort the cells and perform live cell microscopy on these.

This has been verified in follow-up experiments from our group (Suárez-Cortés, manuscript in preparation), where in three independent experiments (N = 3, n = 200), all salivary gland sporozoites were confirmed to be GFP positive by live-cell fluorescence microscopy. In Figure S2, the GFP positive sporozoites form a distinct population with clear separation from the GFP negative background.

In addition, in a preprint from our group (Suárez-Cortés et al., 2023), it was shown that the GFP-positive population was also positive for anti-CSP antibodies, which is a well-established sporozoite surface marker and further confirms that the analyzed population indeed consists of sporozoites. We have added a paragraph to the discussion section of the revised manuscript to reflect this point (lines 373-377 in Revised Manuscript with Track Changes).

Attachment Submitted filename: Response to Reviewers.docx

# Peer review: Decision Letter 1

**DOI:** 10.1371/journal.pone.0354060.r003

**Authors:** Shahin Tajeri

7 Oct 2026

Identification of *Plasmodium*  sporozoites using angle-resolved scattered light pulse shapes

PONE-D-26-30806R1

Dear Dr. Levashina,

We’re pleased to inform you that your manuscript has been judged scientifically suitable for publication and will be formally accepted for publication once it meets all outstanding technical requirements.

Within one week, you’ll receive an e-mail detailing the required amendments. When these have been addressed, you’ll receive a formal acceptance letter and your manuscript will be scheduled for publication.

An invoice will be generated when your article is formally accepted. Please note, if your institution has a publishing partnership with PLOS and your article meets the relevant criteria, all or part of your publication costs will be covered. Please make sure your user information is up-to-date by logging into Editorial Manager at Editorial Manager® and clicking the ‘Update My Information' link at the top of the page. For questions related to billing, please contact billing support.

If your institution or institutions have a press office, please notify them about your upcoming paper to help maximize its impact. If they’ll be preparing press materials, please inform our press team as soon as possible -- no later than 48 hours after receiving the formal acceptance. Your manuscript will remain under strict press embargo until 2 pm Eastern Time on the date of publication. For more information, please contact onepress@plos.org.

Kind regards,

Shahin Tajeri, D.V.M. Ph.D.

Academic Editor

PLOS One

**Comments to the Author**

Reviewer #1: All comments have been addressed

Reviewer #2: All comments have been addressed

**********

2. Is the manuscript technically sound, and do the data support the conclusions?

Reviewer #1: Yes

Reviewer #2: Yes

**********

3. Has the statistical analysis been performed appropriately and rigorously?

Reviewer #1: Yes

Reviewer #2: Yes

**********

4. Have the authors made all data underlying the findings in their manuscript fully available?

The PLOS Data policy

Reviewer #1: Yes

Reviewer #2: Yes

**********

5. Is the manuscript presented in an intelligible fashion and written in standard English?

Reviewer #1: Yes

Reviewer #2: Yes

**********

Reviewer #1: The authors have answered most of the queries and comments made by both reviewers. In my view this version is in a format that can be accepted for publication.

Reviewer #2: I thank the authors for adressing my comments and recomment the manuscript for publication.

I would suggest to include the PCA analysis into the manuscript as a supplement, as I think it does support the work of the authors in suggesting that the predicited classification via temporal analysis is supperior to a simple PCA analysis. This could of course also be expanded in future work by testing prediction rates via PCA alone, and testing if it is possible to encode the temporal information into the PCA in a meaningful way to improve predictions.

**********

what does this mean?). If published, this will include your full peer review and any attached files.

If you choose “no”, your identity will remain anonymous but your review may still be made public.

**Do you want your identity to be public for this peer review?**  For information about this choice, including consent withdrawal, please see our Privacy Policy

Reviewer #1: No

Reviewer #2: No

**********

# Editor report: Acceptance letter

**DOI:** 10.1371/journal.pone.0354060.r004

**Authors:** Shahin Tajeri

PONE-D-26-30806R1

PLOS One

Dear Dr. Levashina,

I'm pleased to inform you that your manuscript has been deemed suitable for publication in PLOS One. Congratulations! Your manuscript is now being handed over to our production team.

At this stage, our production department will prepare your paper for publication. This includes ensuring the following:

* All references, tables, and figures are properly cited

* All relevant supporting information is included in the manuscript submission,

* There are no issues that prevent the paper from being properly typeset

You will receive further instructions from the production team, including instructions on how to review your proof when it is ready. Please keep in mind that we are working through a large volume of accepted articles, so please give us a few days to review your paper and let you know the next and final steps.

Lastly, if your institution or institutions have a press office, please let them know about your upcoming paper now to help maximize its impact. If they'll be preparing press materials, please inform our press team within the next 48 hours. Your manuscript will remain under strict press embargo until 2 pm Eastern Time on the date of publication. For more information, please contact onepress@plos.org.

You will receive an invoice from PLOS for your publication fee after your manuscript has reached the completed accept phase. If you receive an email requesting payment before acceptance or for any other service, this may be a phishing scheme. Learn how to identify phishing emails and protect your accounts at https://explore.plos.org/phishing.

If we can help with anything else, please email us at customercare@plos.org.

Thank you for submitting your work to PLOS One and supporting open access.

Kind regards,

PLOS One Editorial Office Staff

on behalf of

Dr. Shahin Tajeri

Academic Editor

PLOS One
